# Fire–Moisture Interactions Shape Recovery Trajectories Across Temperate Vegetation Communities

**DOI:** 10.1111/gcb.71057

**Published:** 2026-08-13

**Authors:** Emily K. Simpson, Glenda M. Wardle, Christopher R. Dickman, Aaron C. Greenville

**Affiliations:** ^1^ School of Life and Environmental Sciences The University of Sydney Sydney New South Wales Australia

**Keywords:** 2019/20 bushfires, drought, extreme precipitation, fire regimes, remote‐sensing, vegetation recovery

## Abstract

The recovery of vegetation following fire is increasingly shaped by concurrent climate‐driven hydrological extremes, such as drought and extreme precipitation. Under these multi‐disturbance scenarios vegetation communities are likely to show different recovery thresholds and trajectories of change, but this is yet to be fully understood or quantified. We used the Greater Blue Mountains World Heritage Area in south‐eastern Australia as a model system to assess how sustained moisture surplus or deficit could differentially influence the recovery of forest, heath and wetland vegetation from low‐extreme severity fire. From 2015 to 2024, this region experienced severe drought, extensive bushfires (the ‘Black Summer’ bushfires) and extreme rainfall events. By using a new remote‐sensing index (the Post‐fire Stability Index) that measures the rate of change in a disturbed system, we found that most vegetation communities could recover following low‐severity fire even in the case of poor soil moisture conditions, but moisture became increasingly limiting as burn severity increased. Certain communities, such as wet sclerophyll forests and freshwater wetlands, experienced disproportionate declines (up to 5 times greater than other communities) as burn severity and drought intensified. These communities are likely at greatest risk as climate drying escalates. We stress that future recovery from fire in temperate communities will be governed not only by the characteristics of the fire itself and those of the affected vegetation community, but also by the extent to which these characteristics interact with prevailing hydro‐climatic conditions.

## Graphical abstract

Future recovery from fire will be governed not only by characteristics of the fire itself and those of the affected vegetation community, but also by the extent to which these characteristics interact with prevailing hydro‐climatic conditions. Favourable soil moisture conditions post‐fire increase the likelihood that ecosystems will recover to a stable state, but poor moisture conditions heighten the risk of degradation or collapse. Both moisture relationships are most pronounced when fire severity is high‐extreme. Higher severity fire, followed by drought, is likely to disproportionately impact vegetation types with moisture‐sensitive growth responses, such as freshwater wetlands and wet sclerophyll forests. Thus, these communities are expected to be most vulnerable under future climate drying.

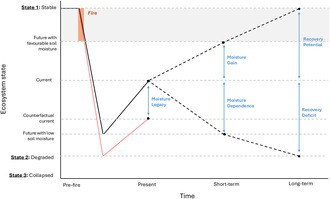

## 1 Introduction

‘Ecological surprises’ (*sensu* Paine et al. 1998) in empirical studies have underscored the wide variability of responses to multiple, interacting disturbance agents (Burton et al. 2020). Responses to individual disturbances, or to regimes of single drivers (e.g., fire regimes; Bowman et al. 2020), are well documented, however the mechanisms by which combinations of disturbances can modify ecosystem structure and function are poorly understood. In terrestrial environments, disturbances such as fire, drought or flooding rains can act synergistically (e.g., Bino et al. 2021; Qiu et al. 2021) or antagonistically (e.g., Batllori et al. 2017; Itter et al. 2019) to drive complex ecological outcomes, distinct from the consequence of each in isolation (Folt et al. 1999). The uncertainty of these outcomes and the timescales over which they occur directly limit our ability to accurately forecast, and thus manage, future ecosystem states.

Recovery trajectories following disturbance are difficult to predict because many contemporary events, or sequences of events, have no observed historical analogue (Milly et al. 2002; Zscheischler et al. 2018). Recovery trajectories can be non‐linear, occur over extended time scales, be contingent on prior ecosystem condition and vary across ecosystem types (Cole et al. 2014; Walden et al. 2022; Spatola et al. 2023). Additionally, as particular disturbances increasingly coincide with other agents for example, wildfires that occur alongside heatwave events or insect outbreaks that follow drought (Turner and Seidl 2023), feedback loops that confer ecological resilience are more likely to be disrupted (Buma 2015; Johnstone et al. 2016). This may push systems beyond recovery thresholds and into novel ecological states (Bergstrom et al. 2021). For instance, in south‐eastern Australia, high frequency fires have resulted in diminished resprouting capacity and subsequent mortality in temperate eucalypt forests (Fairman et al. 2019). Similarly, in North America, compound fire, windstorm and harvesting events drove monotypic stand development in previously diverse forested ecosystems (Busby et al. 2008; Gagnon and Platt 2008). As ecosystem resilience scales broadly with recovery time (Ingrisch and Bahn 2018), a reduction in resilience driven by multiple disturbances is likely to extend recovery timelines, although few studies have explored this directly. Ultimately, understanding this relationship will be key to ensuring the long‐term persistence of ecosystems amid ongoing climate change.

Fire is a major global disturbance agent that is projected to increase in frequency and severity due to its association with climate. Nearly half of the Earth's burnable surface now experiences extreme fire weather (Jain et al. 2021), and this has had consequences for global fire behaviour that include lengthening fire seasons (Jones et al. 2022) and increasing night‐time fire activity (Balch et al. 2022). These changes are likely to extend and alter recovery trajectories, with the magnitude of change appearing to differ among and between ecosystems (e.g., among forests; Hislop et al. 2019a).

In recent years, the impact of fire has been compounded by both pre‐ and post‐fire drought. Prominent examples include the 2017 Portuguese wildfires (Ramos et al. 2023) and the 2018 Californian wildfires (Brown et al. 2020). These synergistic drought‐fire events have driven elevated tree mortality worldwide (van Nieuwstadt and Sheil 2005; Brando et al. 2014; Cansler et al. 2024) and have increased the likelihood of permanent ecosystem state shifts (Lloret et al. 2011; Liu et al. 2022; Volkova et al. 2025). Drought can exert selective pressure on plant traits related to fire resistance (i.e., structural/protective traits) and resilience (i.e., regenerative traits), altering not only the severity of succeeding fire events (e.g., Ramos et al. 2023) but the structure and composition of post‐fire landscapes (Batllori et al. 2018). The combined effect of drought and fire may indeed create distinct environments that would otherwise be observed only under high occurrence of either in isolation (Batllori et al. 2017).

Perhaps unintuitively, fire is also increasingly likely to intersect with extreme rainfall events (Herold et al. 2021; Touma et al. 2022). Precipitation variability in many global regions is influenced by El Niño Southern Oscillation (ENSO) cycles, which are expected to reach extremes more frequently under climate change (Cai et al. 2014, 2015). While these extremes are more often discussed in the context of increasing dryness, they can also drive unusually wet conditions. Excess rainfall in the year preceding fire can increase fire probability by heightening fuel loads (Verhoeven et al. 2020) and in some cases, this effect exceeds that of antecedent drought (Trauernicht 2019). Following fire, exacerbated by changes to soil properties and vegetation cover, high precipitation can then increase the risk of surface run‐off, flooding and debris flow (McGuire and Youberg 2019; Agbeshie et al. 2022; Xu et al. 2023). Whilst rainfall is crucial for post‐fire vegetation recovery, particularly in communities reliant on inundation (Mackay et al. 2024), prolonged periods of high precipitation can drive negative ecological outcomes, particularly if following large, severe wildfire (Xu et al. 2023).

The global rise in extreme fire seasons that coincide with, and/or are driven by, hydrological extremes, is perhaps best illustrated by the 2019/20 bushfire season in south‐eastern Australia. The Black Summer bushfires were preceded (and in large part, driven) by a severe multi‐year drought that resulted in mass canopy die‐back across Australia's eucalypt forests (Nolan et al. 2020; Nolan, Gauthey, et al. 2021; Devanand et al. 2024). It was then an exceptional rainfall event that brought the fires to conclusion; the final stage in a catastrophic sequence of disturbances that saw ∼19 Mha of vegetation burn and ∼3 billion animals killed or displaced (Filkov et al. 2020; van Eeden et al. 2020). The likelihood of such compound events re‐occurring in the region is high given the rise in observed temperatures (0.5°C per decade since 1990), reduction in cool‐season rainfall (9% decline since 1994) and projected increase in daily rainfall extremes (25% increase under a high emissions scenario) (CSIRO and BOM 2024; Zhu et al. 2024). Some vegetation communities, particularly those supporting resprouting eucalypts, may be able to cope with this shift toward more frequent (Collins 2020; Bendall et al. 2022) and severe (Haslem et al. 2016; Collins et al. 2021) wildfire. Some may also persist despite increasing drought incidence through a variety of physiological adaptations such as low rates of photosynthesis and stomatal conductance (Vilagrosa et al. 2014). However, the effect of fire coinciding with either moisture extreme is likely to increase the risk of declining community resilience, particularly in fire‐sensitive communities. These effects have been observed in Australian forests (Bendall et al. 2022; Walden et al. 2022; Xu et al. 2023; Bendall, Collins, et al. 2024), although have been less explored in other vegetation communities (*but see* Mackay et al. 2024; Stanton et al. 2025). Given the sheer scale of Black Summer and other recent wildfire events (e.g., the 2023 Canadian wildfires; Jain et al. 2024), accurate post‐fire assessment now requires datasets with broad temporal and spatial resolution that can effectively account for hydroclimatic extremes.

Satellite‐derived spectral indices provide a means of characterising recovery trajectories at a scale relevant to land managers and policy. Field‐based assessment of post‐fire forests and wetlands in Australia show strong correlation to indices such as the Normalised Burn Ratio (NBR) or Normalised Difference Vegetation Index (NDVI), which can be derived from a variety of sensors (Sentinel, Landsat or MODIS) (Caccamo et al. 2014; Tran et al. 2018; Anzooman et al. 2024). Post‐fire literature is dominated by methods comparing these indices relative to a pre‐fire baseline, including in areas heavily affected by Black Summer (e.g., Gibson and Hislop 2022; Verdon‐Kidd and Gibson 2025). However, the increasing frequency of severe weather events, coupled with broader shifts in disturbance regimes, make adequate assessment of baseline conditions complex (Gibson et al. 2022), even when introducing longer time series and trend‐fitting to characterise trajectories before and after fire (e.g., Hislop et al. 2019b). Further, given post‐fire recovery is rarely linear, there is the likelihood that post‐fire spectral values exceed a pre‐fire benchmark before an ecosystem has truly stabilised, misrepresenting time‐to‐recovery. This is particularly likely if fire is followed by favourable hydroclimatic conditions. To address these concerns, the Post‐fire Stability Index (PFSI), based on a modified Normalised Burn Ratio (NBR2) that contrasts short‐wave infra‐red (SWIR) bands, proposes a comparison of fire‐affected areas to contemporary unburnt regions (Gibson et al. 2022). NBR2 has previously been suggested as a powerful candidate for post‐fire monitoring in south‐eastern Australia due to the sensitivity of SWIR wavelengths to forest moisture and structure (Hislop et al. 2018). The PFSI uses annual timesteps to track the rate of change in a burnt system, defining recovery as the point at which the system stabilises and is synchronous with the unburnt environment (Gibson et al. 2022). It shows strong correlation to field metrics such as resprouting, canopy cover and foliage projective cover. Overall, the PFSI allows for recovery to be measured independent of pre‐fire condition; a key advantage in a rapidly changing and unpredictable climate.

Here, by measuring change in the PFSI over a 10‐year period, we aimed to assess how sustained moisture surplus or deficit could differentially influence the recovery of forest, heath and wetland vegetation from low‐extreme severity fire. Few papers are yet to assess the fire‐moisture relationship across multiple vegetation types in the same climate niche, and to our knowledge none have done so using this remote‐sensing index.

We used the Greater Blue Mountains World Heritage Area (GBMWHA: 1,032,649 ha; NPWS and Environment Australia 2000) in south‐eastern Australia as our model system. During the Black Summer, seven bushfires burnt 79% of the GBMWHA; an unprecedented 29% of which was burnt at high‐extreme severity (Smith and Smith 2022). The largest of these fires, the Gospers Mountain fire, was the largest single‐ignition fire in Australia's recorded history (Filkov et al. 2020; State Government of NSW 2020). The sheer extent of these fires was attributed to the preceding drought (Nolan et al. 2020). Temperatures and vapour pressure deficits were abnormally high and cool season rainfall deficits were ∼−50% over three consecutive years, leading to increasing terrestrial water deficits and extreme plant and soil dryness (Nolan, Bowman, et al. 2021; Devanand et al. 2024). Both drought and fire were brought to a close in February 2020 by a heavy rainfall event, although this too had acute impacts on vegetation through flash flooding and landslide events. Rainfall remained above average in the region for a further 3 years. We used this sequence of disturbances to predict that: (a) fire severity would mediate the effect of moisture on recovery; and (b) this interaction would differ among vegetation communities. We expected community response to fire would diverge most strongly at either end of the moisture spectrum given community variation in fire tolerance, regeneration strategies and water needs. We concluded by applying these modelled relationships to the post‐fire moisture conditions following Black Summer, examining how these relationships translated into community‐specific recovery trajectories over a five‐year period. Overall, we show how our results can inform assessment of current ecosystem condition and be used to infer future vulnerability under interacting fire and moisture extremes.

## 2 Materials and Methods

### 2.1 Study Region

The GBMWHA is located in New South Wales (NSW), Australia (32.37°–34.38° S, 149.90°–151.12° E). It is a deeply incised sandstone tableland dominated by temperate eucalypt forest, with pockets of rainforest, woodland, heathland and wetland (see Table S1). The GBMWHA boasts over 1500 plant species, or 10% of Australia's vascular plants, many of which are threatened, endemic and/or evolutionarily relict (Hammill and Tasker 2010). Soils in the GBMWHA are often dry and infertile, but pockets of rich volcanic and clay soils persist, particularly in flatter areas along eroded ridges or footslopes (DEC 2006). Elevation in the area spans 10 m to 1300 m above sea level, with mean minimum and maximum temperatures of 8°C and 18°C respectively (average of Mt. Boyce, Oberon and Nello Mountain weather stations; BOM 2026). Mean annual rainfall is just under 1000 mm, noting that areas to the north‐east are drier and in rainshadow (Cockerill et al. 2013).

### 2.2 Site Selection

**FIGURE 1:**
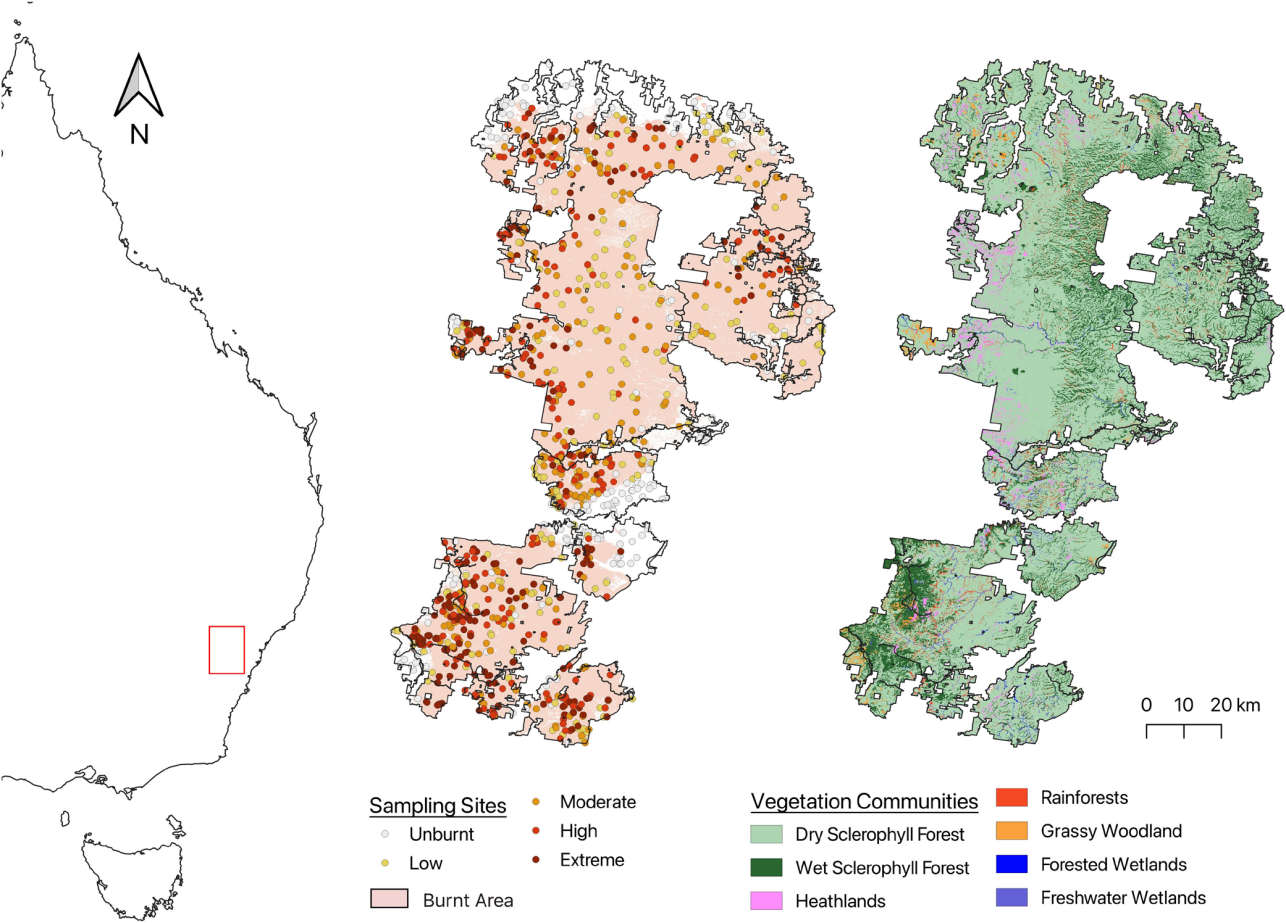
Location of the study area in south‐eastern Australia (left). Panels on the right show extent of the 2019/20 bushfires and vegetation communities in the region. Sampling sites mapped by fire severity class, although noting they are also stratified by vegetation community.

Monitoring sites (n = 760) were stratified based on vegetation community type and fire severity, noting that final site numbers were constrained by available satellite imagery (Figure 1). Sites were established with a minimum interpoint distance of 90 m and were sampled seasonally over a 10‐year period: 2015–2024. Vegetation type was classified using the NSW State Vegetation Type Map (SVTM; State Government of NSW and DCCEEW 2020a), which groups plant communities under the formations and sub‐formations distinguished by Keith (2004). Eleven vegetation formations and sub‐formations were identified in the GBMWHA. We selected seven for analysis, combining sub‐formation classifications and excluding grassland and grassy semi‐arid woodland communities due to limited spatial extent (< 45 ha combined; Smith and Smith 2022). The final vegetation formations assessed included: dry sclerophyll forest (DSF), wet sclerophyll forest (WSF), heathland (HL), rainforest (RF), grassy woodland (GW), forested wetland (FoW) and freshwater wetland (FrW).

Fire severity was taken from the NSW Fire Extent and Severity Map (FESM; State Government of NSW and DCCEEW 2020b) which classifies burn severity into five classes based on different levels of canopy consumption and vegetation scorch: Unburnt, Low, Moderate, High and Extreme (see Table S2; Gibson et al. 2020). To enable direct comparative sampling, the SVTM and FESM datasets, originally at a 5 m and 10 m resolution respectively, were resampled to match the 30 m resolution of our spectral index; the PFSI (Methods 2.4).

### 2.3 Soil Moisture Data

Soil moisture values were obtained from the Soil Moisture Integration and Prediction System (SMIPS) developed by the Terrestrial Ecosystem Research Network (TERN; Stenson et al. 2021). SMIPS is informed by precipitation and evapotranspiration data from the Bureau of Meteorology's Australian Water Resource Assessment Model (AWRA), physical properties from the Soil and Landscape Grid of Australia and on‐ground soil moisture measurements collected from various locations across Australia. It also links daily satellite soil moisture data from the European Space Agency (Stenson et al. 2021).

SMIPs produces proportional percentage estimates of moisture in the top 90 cm of the soil profile, which represents root zone moisture. These estimates are generated daily at a 1‐km^2^ resolution. We aggregated six raster files (two per month at set dates: 10th and 20th) into seasonal composite images over the 10‐year sampling period, averaging moisture estimates per pixel for later data extraction. Although outside our direct sampling period, composite images were also generated for 2014 as we anticipated a lagged vegetation response to soil moisture. All composite image creation was done in QGIS v. 3.38.0 (QGIS Development Team 2024).

### 2.4 The Post‐Fire Stability Index

PFSI values were generated from Landsat 8 Operational Land Imager images (30 m pixel size), downloaded as Level 2 surface reflectance products on Google Earth Engine. Surface reflectance was standardised to nadir viewing geometry with a fixed 45° solar zenith angle to reduce anisotropic reflectance effects. The bi‐directional reflectance distribution function (BRDF) adjustment was implemented using RossThick–LiSparse kernels (Roy et al. 2016) and included a terrain illumination correction based on a 30 m digital elevation model derived from the Shuttle Radar Topography Mission (SRTM). Canopy structural effects were incorporated by modifying the geometric scattering kernel with a canopy height model and vegetation‐specific crown radius parameters. This meant that shadowing effects were parameterised differently for tall, dense communities compared to short, open vegetation types.

Corrected images were combined into seasonal composites for each target year (2015–2024), with the summer season defined as December of the previous year through to February of the target year. While the original methodology used images only from the immediate post‐fire season, we generated composites across the full year to increase our sample size and to ensure that PFSI values could be directly compared across the annual range of soil moisture conditions. PFSI values for each pixel were calculated from:

$$\mathrm{NBR}2=\frac{\left(\text{SWIR}1-\text{SWIR}2\right)}{\left(\text{SWIR}1+\text{SWIR}2\right)}$$

$$\text{PFSI}=\frac{\mathrm{NBR}2\;\mathrm{at}\;\text{time}\;\left(t\right)-\mathrm{NBR}2\;\mathrm{at}\;\text{time}\left(t-1\right)}{\sqrt{\left|\frac{\mathrm{NBR}2\;\mathrm{at}\;\text{time}\;\left(t-1\right)}{1000}\right|}}$$

We note that although SWIR can produce exaggerated signals in wetland environments due to moisture sensitivity, the PFSI, which measures change relative to the previous year, mitigates these effects by normalising interannual differences.

PFSI values are processed using a standardised classification table to indicate ranges of stability or fluctuation (Table 1). Vegetation recovery is achieved when PFSI values are approximately 0 (± 50), and are synchronous with the unburnt environment over multiple years.

**TABLE 1:** Standardised classification table for Post‐fire Stability Index (PFSI) values. As in Gibson et al. (2022).

PFSI values	Vegetation change
>500	Extreme increase
400to500	Very large increase
300to400	Large increase
200to300	Moderate increase
100to200	Small increase
50to100	Very small increase
−50to50	Stable
−50to−100	Very small decrease
−100to−200	Small decrease
−200to−300	Moderate decrease
−300to−400	Large decrease
−400to−500	Very large decrease
<−500	Extreme decrease

### 2.5 Statistical Analysis

Data at each site were extracted seasonally from the stack of generated rasters that contained PFSI and soil moisture values. We used a Bayesian generalised additive mixed model (GAMM) fitted with the *brms* package in R v. 4.5.0 (R Core Team 2025) to model PFSI by fire severity, vegetation community and soil moisture, including all two‐ and three‐way interactions. The model also included a spline for the smooth effect of year fitted separately for each fire severity class, to visualise non‐linear recovery trajectories. A random intercept for site was included to account for among‐site differences. The full model can be understood as:

$$\begin{array}{c} \text{PFSI}\;\sim \;\text{Fire Severity}*\text{Vegetation Community}*\text{Soil Moisture}\\ +s\;\left(\text{Year},\mathrm{by}=\text{Fire Severity}\right)+1\;|\;\text{Site} \end{array}$$

where PFSI is the dependent variable (the relative change in vegetation biomass), Fire Severity and Vegetation Community are categorical predictors and Soil Moisture is a continuous covariate. Year is a continuous temporal predictor modelled with smooths and Fire Severity a factor interacting with the spline to allow for different smooth shapes. A basis dimension of *k* = 6 was used for each smooth and visual inspection confirmed they were not excessively wiggly and biologically plausible. Site is a random effect term to account for repeated measures and grouping by sampling location. Similar to a before‐after‐control impact study, some sites remained unburnt throughout the study period (acting as a controlled baseline) whilst others burnt at varying severities.

Soil moisture was standardised and modelled with a yearly lag to account for delayed vegetation response to root‐level moisture availability. Lag length was confirmed by comparing models with no lag, a seasonal lag and a yearly lag using Akaike's Information Criterion for model selection (Table S3; Akaike 1974). The model was fitted with weakly informative and regularising priors and a Student's *t* distribution to account for potential heavy‐tailed residuals. We ran four Markov Chain Monte Carlo (MCMC) chains with 6000 iterations, including a 2000 iteration warm‐up. Parameter estimates were regarded as significant if their 95% credible intervals did not include zero. MCMC convergence was assessed using the $\hat{R}$ statistic, with values below 1.01 considered evidence of adequate convergence.

Where appropriate, soil moisture was grouped into five categories based broadly on percentiles outlined by the Australian Combined Drought Indicator (CDI; Table 2). The full CDI integrates multiple indicators of drought (rainfall, soil moisture, evapotranspiration and vegetation greenness; Guillory et al. 2023). However for the purpose of this study, we focused solely on soil moisture as a direct reflection of the water available for plant growth following fire. Indirectly, soil moisture values should also reflect variation in precipitation, evapotranspiration and vegetation greenness.

**TABLE 2:** Australian Combined Drought Indicator (CDI) categories used to visualise model predictions.

CDI category	Percentile range	Absolute soil moisture values (%)
Extreme drought: No soil moisture available to plants. Water bodies nearly empty or dry.	2nd−5th	∼6−10
Moderate drought: Soil is dry and plants may brown. Moisture levels in water bodies are low.	10th−20th	∼16−26
Near normal: Soil moisture is appropriate for the time of year.	30th−70th	∼33−65
Moderate wet: Soil is damp and plants are green. Water bodies are fuller than normal.	80th−90th	∼73−81
Extreme wet: Soil is wet. Ground may be saturated and/or flooded. Water bodies are above normal.	95th−98th	∼86−91

*Note:* Categories and their descriptions based on Guillory et al. (2023). Absolute soil moisture values are the range of values present in our dataset.

## 3 Results

The posterior distribution was successfully sampled with $\hat{R}$ = 1 for all parameters, indicating good convergence of all four chains (Table S4). We report posterior means and 95% credible intervals (CrI) for model parameters, and the posterior predicted means where appropriate.

### 3.1 Fire Severity Mediates the Effect of Moisture on Recovery

**FIGURE 2:**
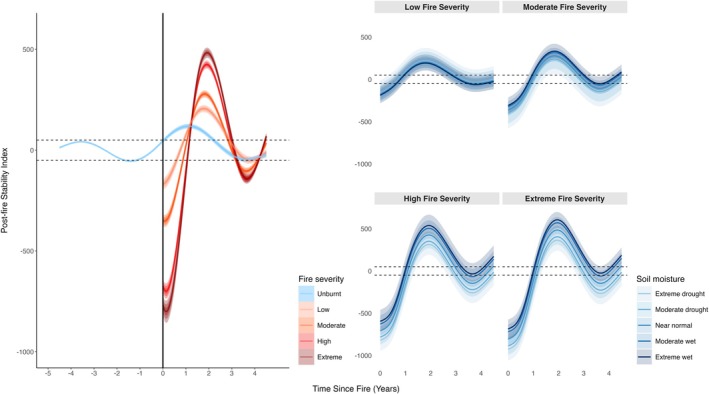
Recovery trajectories of vegetation burnt at low, moderate, high and extreme severity across the Greater Blue Mountains World Heritage Area, Australia, 2015–2024 (left). Each fire severity plotted under five categories of relative soil moisture (right). Categories based broadly on the Australian Combined Drought Indicator: Extreme drought (values 2nd–5th percentile; absolute soil moisture 6%–10%), Moderate drought (10th–20th percentile; 16%–26%), Near normal (30th–70th percentile; 33%–65%), Moderate wet (80th–90th percentile; 73%–81%), Extreme wet (95th–98th percentile; 86%–91%). Dotted lines indicate range of stable vegetation values. Values above the dotted line indicate vegetation has increased from the previous year; values below the dotted line indicate vegetation has declined relative to the previous year. Ribbons depict 95% credible intervals.

Plotted trajectories showed that fire severity was the dominant driver of divergent recovery outcomes; however soil moisture became an increasingly important factor under high‐extreme burn conditions (Figure 2). This pattern was reflected in the estimated effects of soil moisture, which were limited following low severity fire (posterior mean = +1.8, 95% CrI [−24.3 to +27.9]), increased after moderate severity burns (+31.9 [+8.1 to +55.6]), and were greatest following high (+80.6 [+58.5 to +102.9]) and extreme (+82.9 [+53.3 to +112.7]) fire severities. Model predictions indicated that the difference between extreme wet and extreme drought conditions following high‐extreme severity burns was [+222.894] and [+240.08] units greater respectively. This difference was approximately halved in moderate severity fires ([+110.34] units) and very minor in low severity burns ([+24.43] units).

As a result of this divergence, sites exposed to high or extreme severity fire followed by drought saw a secondary decline of vegetation after the initial post‐fire growth peak. In contrast, sites burnt at low to moderate severity were broadly stable following initial vegetation flush regardless of soil moisture condition (Figure 2; Years 3–4). Divergence between each fire severity class was greatest in the first 2 years post‐fire.

### 3.2 The Interactive Effects of Fire Severity and Soil Moisture Differ Across Vegetation Communities

**FIGURE 3:**
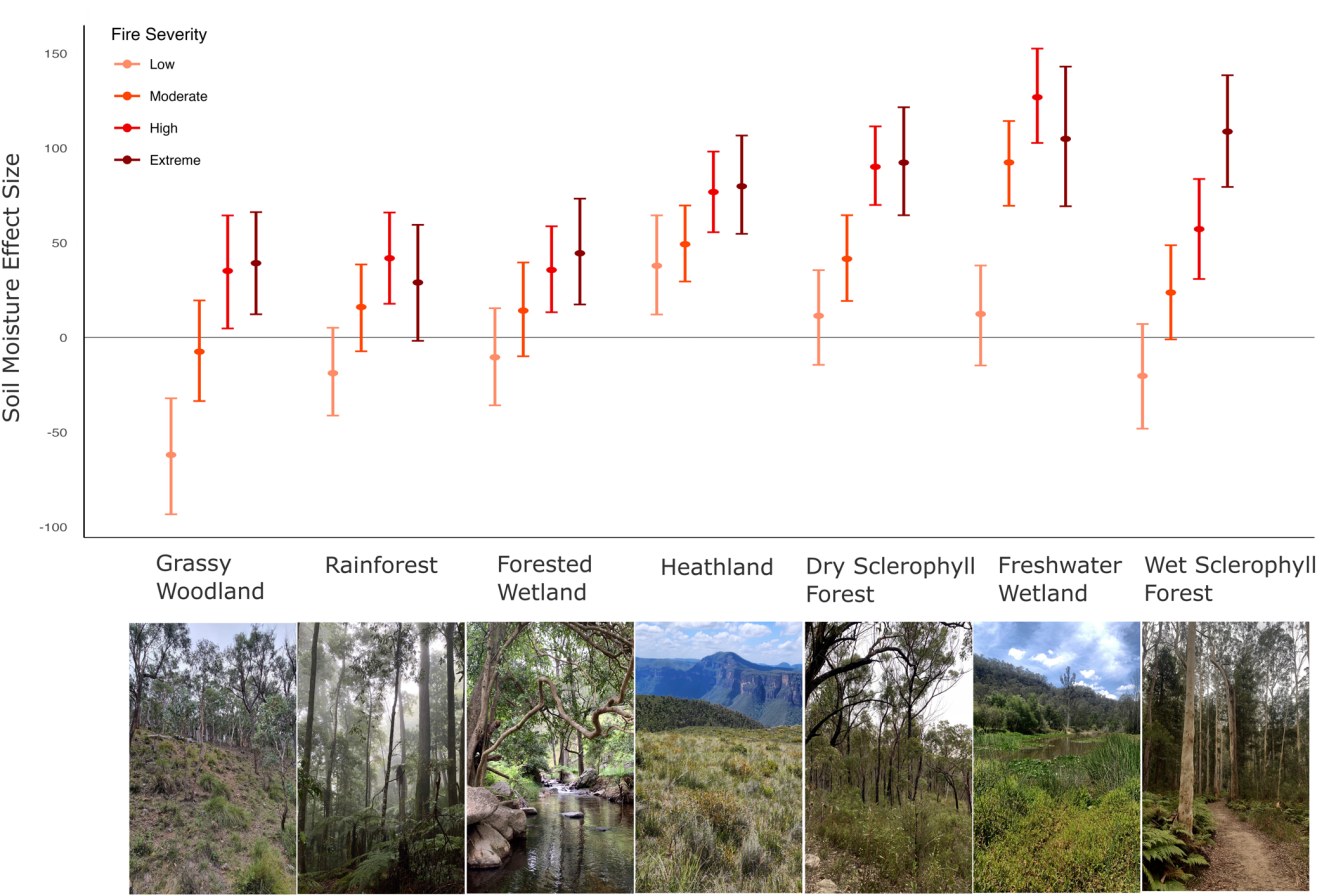
The effect of soil moisture on the Post‐fire Stability Index across seven vegetation communities. Points represent posterior mean estimates and bars represent 95% credible intervals. Positive slopes indicate an increase in post‐fire biomass with increasing soil moisture. Negative slopes indicate reduced biomass with increasing soil moisture. Photo credit: Emily Simpson, Karina Guo & Aaron Greenville.

This severity‐dependent pattern in soil moisture sensitivity was broadly consistent across vegetation communities, though it varied markedly in magnitude (Figure 3; Table S5). At low fire severities, most communities showed negligible soil moisture effects, with the exception of HL and GW which showed small positive (posterior mean = +37.75; 95% CrI [+12.03 to +64.36]) and negative (−62.06 [−93.42 to −32.20]) effects respectively. Following moderate severity burns, only three communities showed positive trends with soil moisture (FrW, HL and DSF). Yet once burns reached high or extreme severity, all communities exhibited a strong growth response to increasing moisture availability. This was particularly pronounced in FrW following high (+126.73 [+102.59 to +152.40]) and extreme (+104.74 [+69.17 to +142.89]) severity fire and in WSF following extreme severity burns (+108.58 [+79.36 to +138.33]). Given soil moisture was modelled as a linear term, these communities were also those that declined the most under extreme drought; FrW and WSF showed a 5× greater decline than the average of all other vegetation communities. The only community to not follow this severity dependent trend was RF. Whilst RF communities showed a positive response to moisture following high severity fire (+41.73 [+17.70 to +65.87]), at extreme severities such effects were negligible (+28.93 [−1.87 to +59.36]). It was the only community with a negligible response to soil moisture at extreme fire severity.

As predicted, differences in recovery outcomes were most pronounced when comparing community response to fire under wet and dry extremes. Under normal soil moisture conditions, all communities show similar relative decline following burns of each severity, diverging by no more than [+37.57] units. However, this divergence was 3–4 fold greater in extreme drought ([+151.48] units) and extreme wet ([+122.65] units). For example, when considering one of the most severe disturbance sequences—an extreme severity fire followed by an extreme drought—GW communities had a PFSI value [+136.52] units greater than FrW. However, had soil moisture conditions remained within the 30th‐70 percentile (i.e., within the range of normal), community responses only differed by [+23.93] units.

### 3.3 Community Recovery Trajectories and Timelines Under Observed Post‐Fire Conditions

**FIGURE 4:**
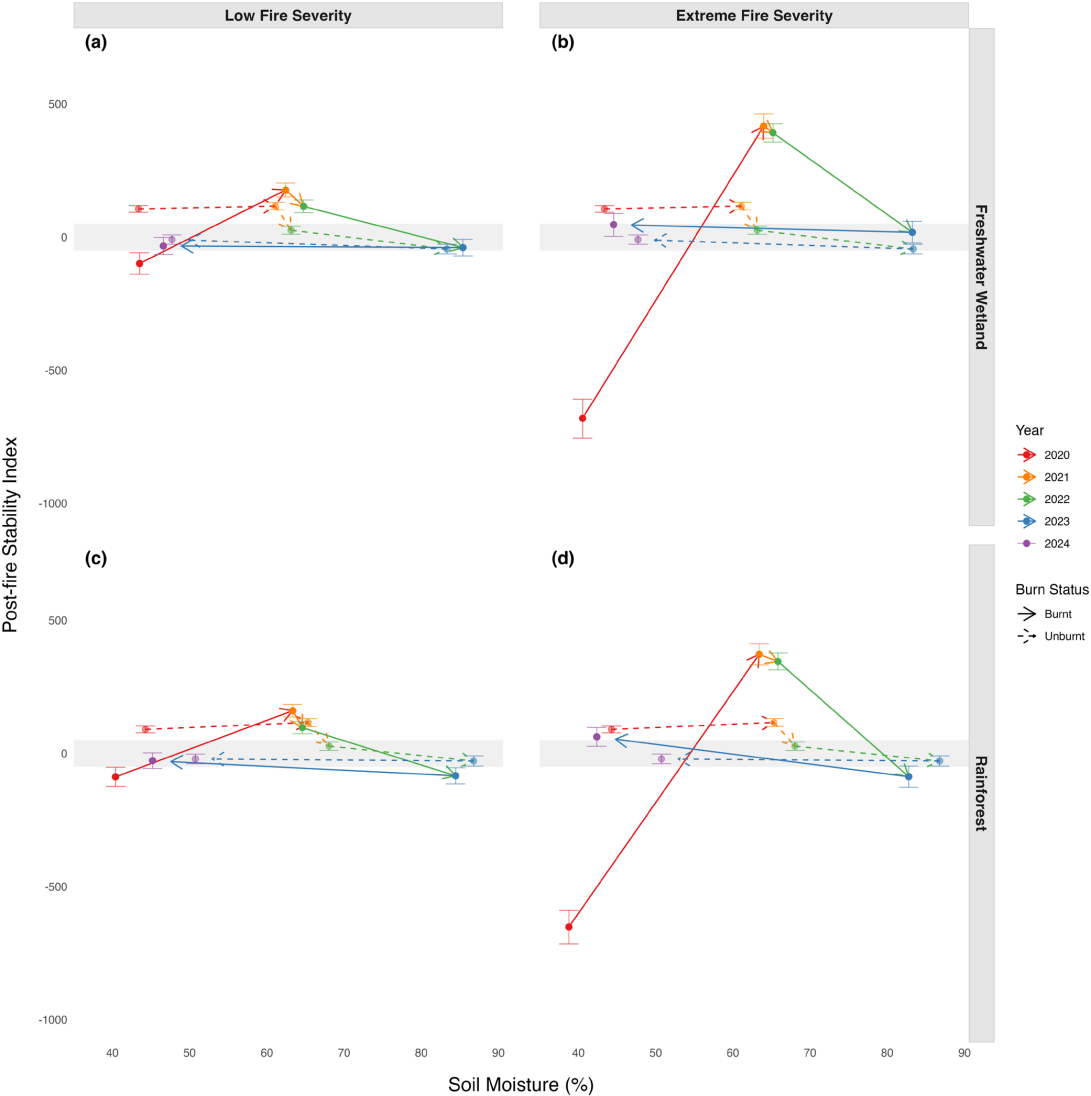
Recovery trajectories in the Greater Blue Mountains World Heritage Area following the 2019/20 bushfire season: (a) Freshwater Wetland burnt at low fire severity, (b) Freshwater Wetland burnt at extreme fire severity, (c) Rainforest burnt at low fire severity and (d) Rainforest burnt at extreme fire severity. Grey shaded regions highlight stable vegetation range. Arrows indicate direction of change and bars depict 95% credible intervals.

The effect of sustained moisture surplus following the Black Summer bushfires was evident in trajectories of vegetation communities burnt at low‐extreme severity (representative trajectories highlighted in Figure 4, Figures S1–S4 for complete outputs).

Following low severity fire, most vegetation communities (HL, DSF, WSF, FrW, FoW) were stable (PFSI values −50to50) and synchronous with unburnt controls by 2023. These conditions were maintained through to 2024, consistent with recovery from fire within ∼3 years. Notable exceptions to this trend were GW and RF, both of which declined in 2023 in response to the exceptional moisture surplus the previous year. Both communities had stabilised by 2024, although additional years of data are needed to determine if this stability is persistent and thus indicative of recovery.

At moderate fire severities, trajectories were more variable. HL and DSF reflected values consistent with recovery by 2023 (i.e., a similar 3 year recovery window), and FrW appeared stable by 2024 (again, noting the need for additional years of data). However, remaining communities continued to fluctuate. GW, RF, FoW and WSF (although marginally), declined in 2023 and failed to stabilise the following year, thus failing to meet recovery criteria within the 5‐year sampling period.

Similarly, no community burnt at high severity met recovery criteria within a 5 year timeframe. DSF, HL and FrW both showed stable values in the final 2 years of sampling but did not maintain convergence with unburnt controls for more than a year. The shape of their trajectories also diverged; DSF and HL communities stabilised in 2023 but increased the following year. FrW increased in 2023 but stabilised in 2024.

Recovery was evident among some communities exposed to extreme severity fire. HL, FrW and WSF showed PFSI values consistent with recovery by 2023. DSF too showed early signs of recovery, albeit with minor fluctuation in PFSI in the final sampling year. In contrast, RF, GW and FoW reflected similar trajectories as under high severity fire. They did not meet the criteria for recovery within the five‐year sampling period.

## 4 Discussion

The effect of sustained soil moisture surplus or deficit on post‐fire recovery was dependent on burn severity. Most vegetation was likely to recover from low severity fire regardless of the moisture conditions that followed; however, recovery from high‐extreme severity burns was contingent on access to soil moisture reserves. This relationship held true for all vegetation communities, although moisture reliance was most pronounced in freshwater wetland and wet sclerophyll forest. Below, we discuss the future resilience of temperate communities under interacting fire–moisture extremes and the implications of our results for management under a changing climate.

### 4.1 Resilience of Vegetation Communities Following Future Fire‐Moisture Extremes

Our results aligned with previous studies in temperate climates that examined the impact of moisture on post‐fire recovery using spectral indices. For example, Pérez‐Cabello et al. (2026) found fire severity and moisture availability were dominant drivers of recovery, particularly during the first 2 years. Pan et al. (2025) further showed that the effect of moisture surplus was greatest at higher fire severities, perhaps due to a transient increase in nutrient and light availability (Carter and Foster 2004; Marod et al. 2004), removal of competitors (Driscoll et al. 2024) and/or the strong ability of many Australian trees and shrubs to resprout, germinate and photosynthesise (Knox and Clarke 2006; Pausas and Keeley 2017). Precipitation, and by extension soil moisture, has previously been suggested as the dominant factor driving structural recovery in Australia (Byrne et al. 2021; Qin et al. 2022), and it is likely that only low moisture thresholds (i.e., only small deviations above the mean) are needed to support regeneration (Pan et al. 2025).

Our results are also consistent with work suggesting freshwater wetland and wet sclerophyll forest are moisture‐sensitive, particularly following high‐extreme severity fire. Post‐fire vegetation recovery in wetland environments is primarily linked to soil moisture fluctuation, both within south‐eastern Australia (Mackay et al. 2024; Anzooman et al. 2024) and globally (Salvia et al. 2012; Kominoski et al. 2021). Evidence suggests that WSF too supports vegetation that can capitalise on soil moisture; for example, resprouting species with post‐fire seedling recruitment (Campbell and Clarke 2006). However, such sensitivities also suggest that the threat of decline following a fire‐drought sequence in these vegetation types is high. We have shown that the magnitude of this decline depends on fire and drought severity, but it is also likely to depend on drought duration and frequency. Ecosystems that experience prolonged and frequent drought take longer to recover and can experience legacy effects that trigger mortality and potential transition to novel states (Allen and Breshears 1998; Jiao et al. 2021; Yao et al. 2023; Volkova et al. 2025). For example, there is concern that a drier, and subsequently more frequently burnt landscape, will transition wet sclerophyll forest into its dry counterpart (Fensham et al. 2024).

Although outside the direct scope of this study, fire‐drought sequences are also likely to increase future fire risk by reducing vegetation moisture and carbohydrate reserves, and this is unlikely to occur uniformly across vegetation communities. One suggestion is that climate‐limited communities may be at greater risk of increased fire incidence than fuel‐limited communities (Pausas and Ribeiro 2013; McColl‐Gausden et al. 2022). Fire occurrence requires sufficient fuel load and fuel dryness (i.e., two of the four fire ‘switches’; Bradstock 2010). As dangerous fire weather, including drought, increases, productive environments may see their vegetation dry to the point of ignition, increasing fire spread and potentially reducing the fire return interval (McDowell et al. 2018; McColl‐Gausden et al. 2022; Doherty et al. 2024). For example, Fensham et al. (2024) showed that fuel loads in wet sclerophyll forest were double that of rainforest during the drought preceding Black Summer, corresponding to a 60% and 23% burn extent respectively. This mechanism may also explain the severity of fires in areas that historically do not burn, including in areas within the spatial extent of this study; for instance, ~15% of rainforest in the GBMWHA burnt at high‐extreme severity during the 2019/20 bushfires (Smith and Smith 2022). There will be fire frequency thresholds after which community persistence becomes uncertain, and this threshold is likely to be lower in areas with limited evolutionary exposure to fire.

Other uncertainties also remain. Although fire severity and soil moisture appear to be the dominant drivers of recovery for most vegetation types, these factors exist alongside other climate and landscape features (e.g., nutrient availability, solar radiation, distance to unburnt areas). For instance, our results are unlikely to suggest that rainforest is resilient to post‐fire drought at extreme fire severities, and instead that extreme severity fire is interacting with other characteristics of the post‐fire landscape to constrain regeneration (although we note recent acknowledgement of burn resilience in rainforest; Baker et al. 2022). We would urge caution in implementing broad‐scale recovery and management plans without consideration for site‐specific factors. Pérez‐Cabello et al. (2026) drew similar conclusions, showing that the inclusion of random effects substantially improved the fit of post‐fire recovery models.

There is also uncertainty as to how fire–moisture interactions may impact common management strategies, such as controlled burning. Controlled burns are often used as a fuel reduction tool, particularly around major population centres, but they show varied success across vegetation and climate types (Clarke et al. 2022). We suggest a prescribed burn undertaken in a moisture surplus may have reduced temporal effectiveness, given these post‐fire moisture conditions promote rapid regrowth and thus may increase or quickly restore fuel load. In contrast, the same controlled burn in drought conditions could lead to cumulative vegetation stress and, as has been seen previously in Australian forests, lead to acute mortality and delayed tree regrowth (Volkova et al. 2025). There is already a strong body of work suggesting that controlled burns have a short window of efficacy (< 5 years) and are less effective under extreme fire weather conditions (Bradstock et al. 2010; Price and Bradstock 2012; Tolhurst and McCarthy 2016).

### 4.2 Recovery From the Black Summer Bushfires

**FIGURE 5:**
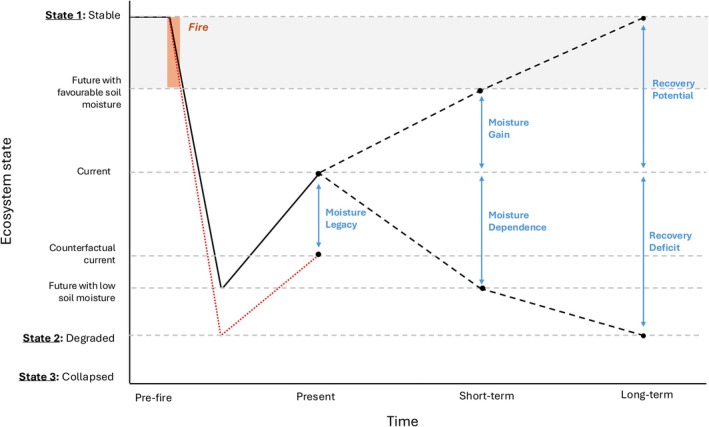
Conceptual diagram of biomass recovery from fire. Adapted from Akçakaya et al. (2018). The solid black line indicates observed post‐fire trajectories to date; dotted red line (counterfactual) indicates modelled trajectories under low soil moisture conditions; dashed black lines indicate predicted future vegetation trajectories. Blue vertical arrows specify the impact of soil moisture: *Moisture legacy*: Impact of favourable soil moisture on post‐fire vegetation growth to date; *Moisture Gain*: Expected short‐term improvement under favourable soil conditions; *Moisture Dependence*: Expected short‐term decline under poor soil conditions; *Recovery Potential*: Likelihood of reaching a stable ecosystem state with prolonged favourable soil moisture; *Recovery Deficit*: Likelihood of ecosystem degradation with prolonged poor soil moisture condition. The magnitude of soil moisture impacts is expected to be greatest in communities with moisture sensitive life history processes, and if following high‐extreme severity fire.

In general, spectral trends across the Sydney basin suggest recovery from fire occurs within 5 to 7 years (Caccamo et al. 2014; Heath et al. 2016). Our trajectories indicate most vegetation types burnt at low severity have recovered within this timeframe. However some communities burnt at higher fire severities have exceeded this 5‐year benchmark, and, given the observed fluctuation in values at the end of our sampling period, may also exceed the upper bound of this recovery window. Longer recovery times following the Black Summer bushfires may be explained by the combination of fire severity and fire extent. High severity, homogenous burns are broadly associated with biodiversity loss (Driscoll et al. 2024; Gibson et al. 2025) and can restrict re‐establishment from unburnt areas as dispersal distance is exceeded (Keane et al. 2008). Long‐term forecasting in eucalypt forest has suggested that exceptional burn severity during the 2019/20 bushfire season will increase recovery time (~15% longer than previous fire years), however the strength of this effect is likely to be lessened due to anomalously high rainfall (Rifai et al. 2024). We too observed this moisture mitigation effect and suggest it is the major driver behind the somewhat favourable condition of fire‐sensitive vegetation types (e.g., freshwater wetlands), even when burnt at extreme severity. We note that the communities that met recovery criteria following extreme severity burns were those we identified as most able to capitalise on excess soil moisture reserves. Overall, future recovery from fire will be governed not only by the characteristics of the fire itself and those of the affected vegetation community, but also by the extent to which these characteristics interact with prevailing hydro‐climatic conditions (Figure 5).

We urge caution with assertions that recovery of biomass following Black Summer was rapid and therefore indicative of ecosystem resilience. Firstly, some studies with these claims (e.g., Qin et al. 2022) had post‐fire recovery timelines < 2 years, which excluded the delayed mortality events reported during later forest field surveys (Volkova et al. 2025). Secondly, many used methods reliant on a pre‐fire baseline, which we re‐assert can under‐estimate recovery times by coupling pre‐ and post‐fire condition. Our methods account for this by comparing burnt sites to contemporary unburnt regions. Finally, rapid regeneration of biomass is not always indicative of an ecosystem returning to its pre‐fire state. High post‐fire precipitation may favour the re‐establishment of certain species due to differences in competitive or adaptive abilities, which could shift the compositional landscape. For example, Volkova et al. (2025) recorded thick layers of *Acacia* following fire, but little to no regeneration of *Eucalyptus* seedlings. Such changes may also elevate future fire risk, particularly in vegetation types where fire spread is dependent on fast‐growing herbaceous fuels (e.g., grassy woodlands). There remains significant uncertainty on the relationship between climate, fire and fuel load and these relationships may change as ecosystems enter novel or transitional states (Keeley and Syphard 2016; McColl‐Gausden et al. 2022). Future climates may facilitate soil drying and thus lower the levels of burnable biomass (particularly in forests; Stegen et al. 2011), but interactions with extreme rainfall events may counteract or alter this trajectory, particularly in vegetation communities whose productivity is moisture‐limited. Ultimately, rapid recovery of biomass does not preclude the possibility that ecosystems have taxonomically or functionally diverged from their original state. Understanding the degree to which this may occur across future fire severities and vegetation types is an important avenue for future research.

### 4.3 Caveats

We acknowledge that spectral recovery is not necessarily indicative of structural, compositional or functional recovery (Celebrezze et al. 2024), although by comparing to contemporary unburnt regions, the PFSI does well to reduce conflating recovery with temporary bursts of understorey vegetation or changes in hydroclimate. The PFSI, and a recent delayed recovery index based on its value range (*see* Gibson et al. 2025), have also been field validated across forested areas in south‐eastern Australia. Sites with delayed recovery showed higher tree mortality and topkill (Bendall, Choat, et al. 2024), and were more likely to have a low incidence of epicormic and basal resprouting (Gibson et al. 2025). However this validation has not been done in wetland or heath communities. We recognise that suggested vegetation mechanisms following fire are inferred and not directly measured in this study. We also note that most mechanistic evidence following compound fire‐moisture disturbance comes from forested environments. We welcome future research that explores physiological responses to fire and moisture extremes in other terrestrial ecosystems. In the context of our study, the PFSI best serves as a tool to prioritise at‐risk vegetation communities for future management, particularly given that field data are rarely available over the spatial and temporal scales needed for effective decision making.

## 5 Conclusion

Our study provides new insight into fire and moisture interactions in temperate vegetation communities. We show that whilst most vegetation is likely to recover from low severity fire regardless of prevailing moisture conditions, recovery from high‐extreme severity burns is contingent on access to soil moisture reserves. Given corresponding evidence that moisture dependence is not uniform across vegetation types, future fire‐drought interactions are likely to drive disproportionate declines in sensitive communities. Here, we have highlighted these declines in freshwater wetland and wet sclerophyll forest, but stress that the predicted increase in fire and drought incidence may exacerbate future declines in other temperate groups. Finally, our findings indicate that whilst fire severity and soil moisture remain dominant drivers of recovery, their interaction with other aspects of the post‐fire system means recovery cannot always be predicted based on these factors alone. We urge caution in implementing broad‐scale management plans that do not account for the complexity of these interactions across vegetation and landscape contexts.

## Author Contributions

**Aaron C. Greenville:** conceptualization, methodology, writing – review and editing, formal analysis. **Christopher R. Dickman:** conceptualization, methodology, writing – review and editing, formal analysis. **Emily K. Simpson:** conceptualization, formal analysis, data curation, methodology, writing – original draft, writing – review and editing, visualization. **Glenda M. Wardle:** conceptualization, methodology, writing – review and editing, formal analysis.

## Funding

This work was supported by Ecological Society of Australia.

## Conflicts of Interest

The authors declare no conflicts of interest.

## Supporting information


**Table S1:** Seven vegetation communities in the Greater Blue Mountains World Heritage Area, as described by Hammill and Tasker (2010). Community extent from Smith and Smith (2022). Photo credit: Emily Simpson, Karina Guo & Aaron Greenville.
**Table S2:** Fire severity classifications as described by Gibson et al. (2020). Canopy scorch describes when leaves are killed but not consumed.
**Table S3:** Model comparison using Akaike's Information Criterion for model selection. Model with yearly moisture lag had the strongest support.
**Table S4:** Posterior means and 95% credible intervals of all model parameters across study sites in the Greater Blue Mountains World Heritage Area, Australia, 2015–2024. Reference levels for fire severity and vegetation type set as Unburnt and Dry Sclerophyll Forest respectively. Effects considered credible and shown in bold if 95% credible intervals do not cross zero.
**Table S5:** Estimated marginal effects (slope) of soil moisture on the Post‐fire Stability Index within each combination of fire severity and vegetation community. Effects considered credible and shown in bold if 95% credible intervals do not cross zero.
**Figure S1:** Recovery trajectories over the period 2020 to 2024 of vegetation communities following low severity fire (solid arrows) compared to unburnt areas (dashed arrows) in the Greater Blue Mountains World Heritage Area. Values representing vegetation stability are shaded in grey. Bars represent 95% credible intervals.
**Figure S2:** Recovery trajectories over the period 2020 to 2024 of vegetation communities following moderate severity fire (solid arrows) compared to unburnt areas (dashed arrows) in the Greater Blue Mountains World Heritage Area. Values representing vegetation stability are shaded in grey. Bars represent 95% credible intervals.
**Figure S3:** Recovery trajectories over the period 2020 to 2024 of vegetation communities following high severity fire (solid arrows) compared to unburnt areas (dashed arrows) in the Greater Blue Mountains World Heritage Area. Values representing vegetation stability are shaded in grey. Bars represent 95% credible intervals.
**Figure S4:** Recovery trajectories over the period 2020 to 2024 of vegetation communities following extreme severity fire (solid arrows) compared to unburnt areas (dashed arrows) in the Greater Blue Mountains World Heritage Area. Values representing vegetation stability are shaded in grey. Bars represent 95% credible intervals.

## Data Availability

The datasets for fire severity and vegetation community type are publicly available on the SEED data portal (https://datasets.seed.nsw.gov.au/dataset/fire‐extent‐and‐severity‐mapping‐fesm‐2019‐20 and https://datasets.seed.nsw.gov.au/dataset/nsw‐state‐vegetation‐type‐map). Soil moisture data is freely available from the Terrestrial Ecosystem Research Network (https://doi.org/10.25901/b020‐nm39). Code used for data processing and extraction are available at: https://doi.org/10.25901/64bm‐jw66.
