# Supplementary material for: Fire–Moisture Interactions Shape Recovery Trajectories Across Temperate Vegetation Communities
Source: Glob Chang Biol. 2026 Aug 13;32(8):e71057. doi: 10.1111/gcb.71057 (PMC13473728; doi:10.1111/gcb.71057)

Table S1: Seven vegetation communities in the Greater Blue Mountains World Heritage Area, as described by Hammill & Tasker (2010). Community extent from Smith & Smith (2022). Photo credit: Emily Simpson, Karina Guo & Aaron Greenville.

Vegetation Community	*Description*	*Image*
Dry Sclerophyll Forest	Open eucalypt forest 10m - 30m tall. Occurs on low-nutrient soils with diverse understories of sclerophyllous shrubs. Covers 72% of the region.	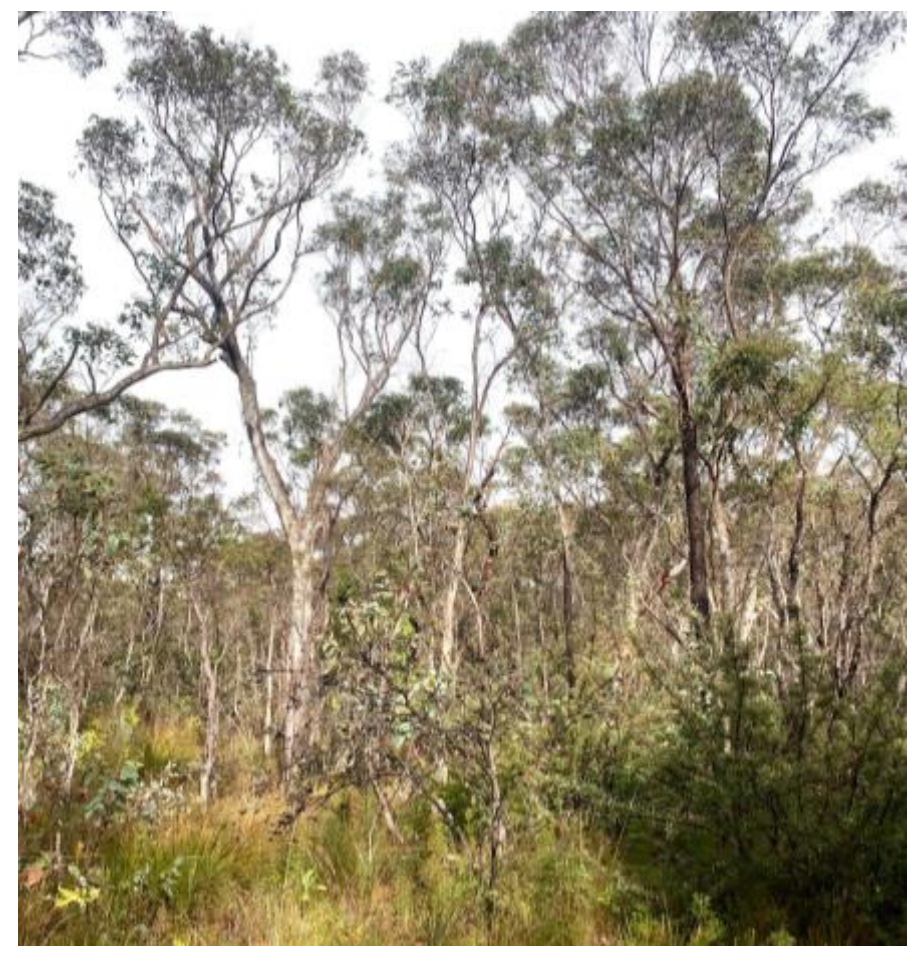
Wet Sclerophyll Forest	Open eucalypt forest reaching up to 70m tall. Occurs on moderately fertile soils with mesic shrubby or grass understorey. Covers 17.7% of the region.	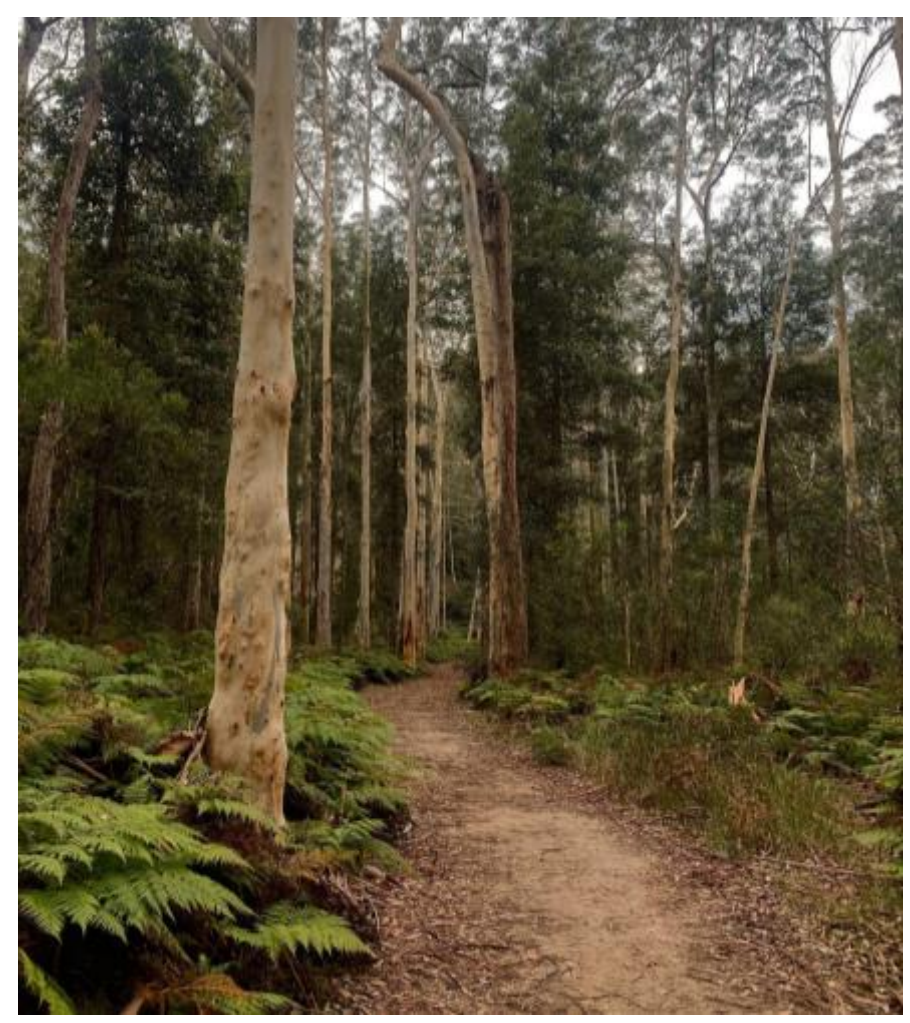

Heathland	Shrubby, treeless communities on shallow, nutrient poor soils. Usually on ridge tops. Covers 3.7% of the region.	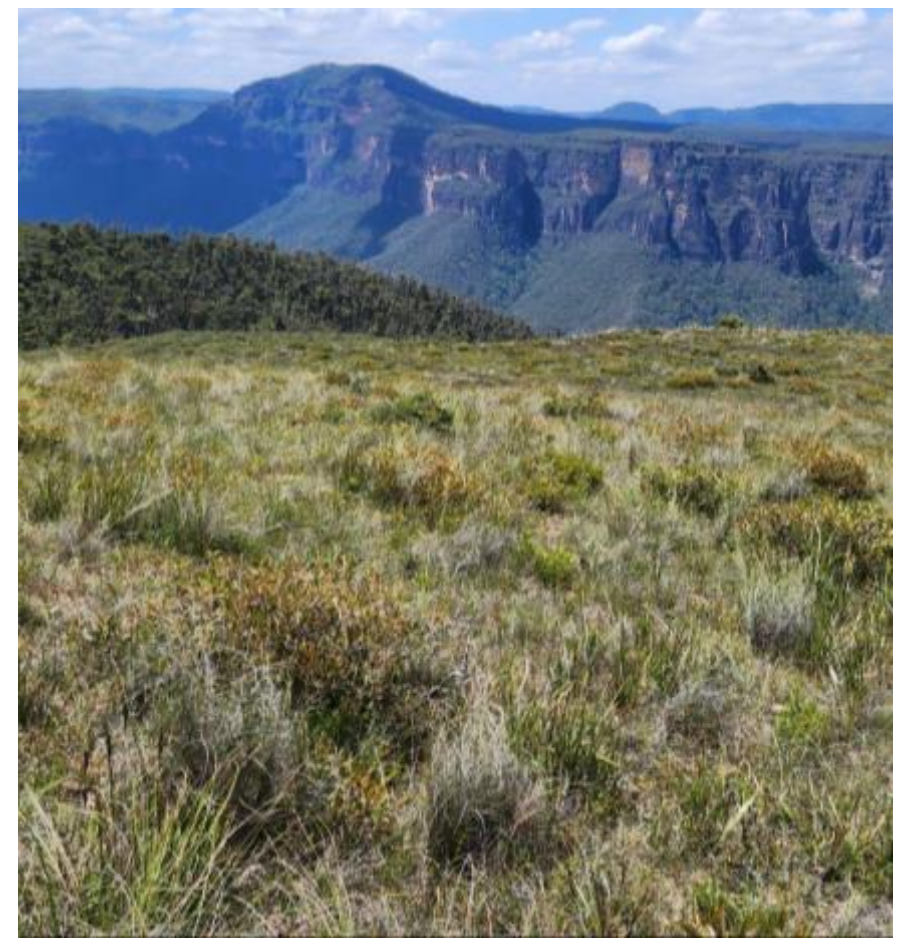
Rainforest	Closed continuous forest on moist, high fertility soil. Shade-tolerant understorey of ferns, herbs and vines. Covers 2.5% of the region.	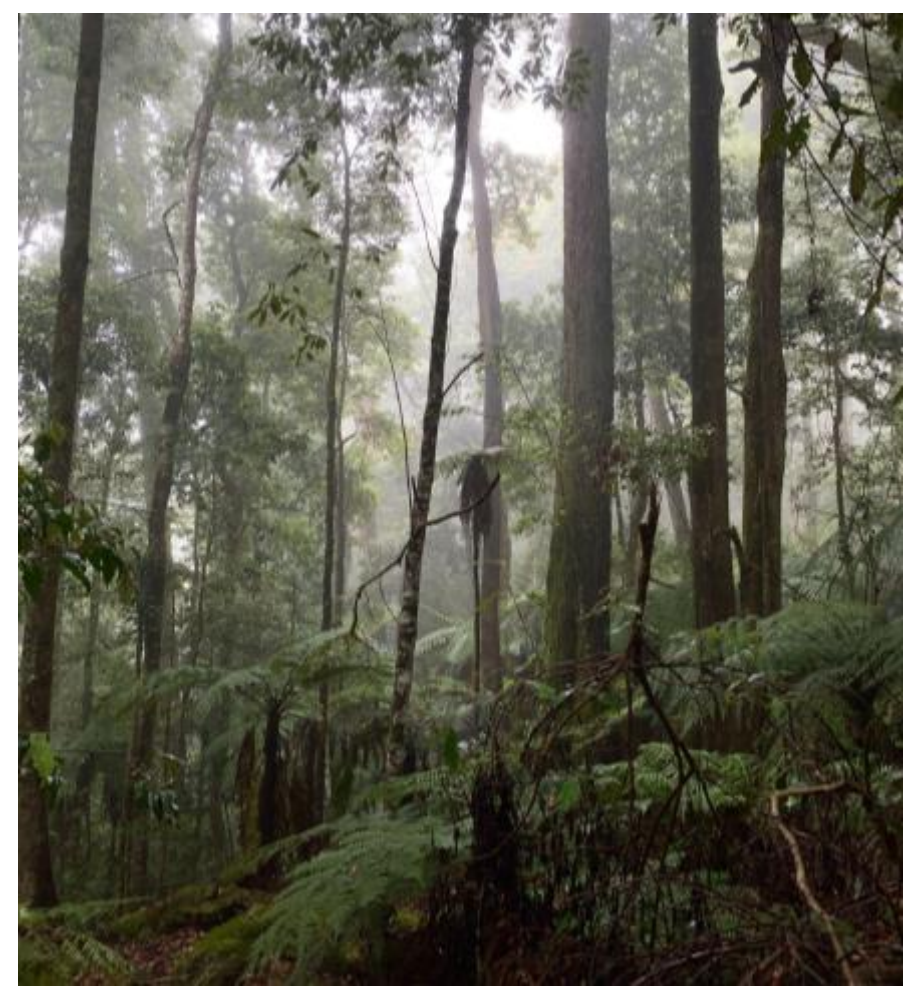
Grassy Woodland	Open eucalypt forest on moderate to high fertility soils. Understorey dominated by grasses and herbs. Covers 1.9% of the region.	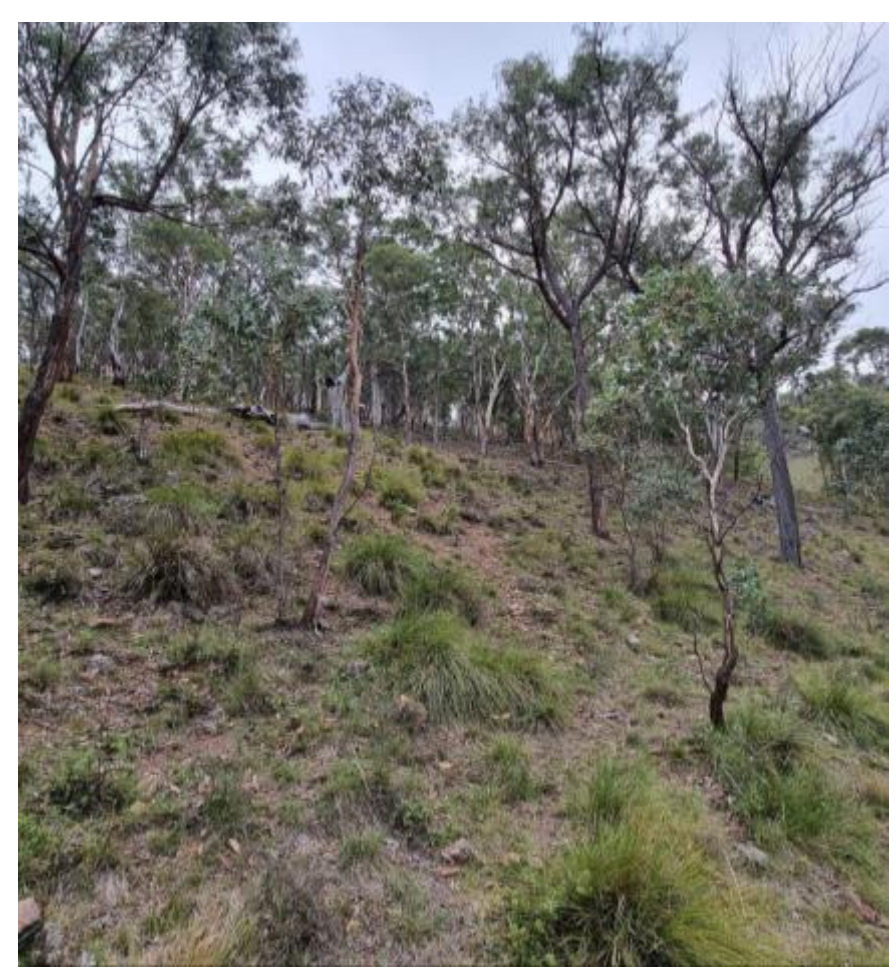

Forested Wetland	Sclerophyllous forest with distinct understorey of grasses, ferns and mesophyllous shrubs. Periodically inundated. Covers 1.3% of the region.	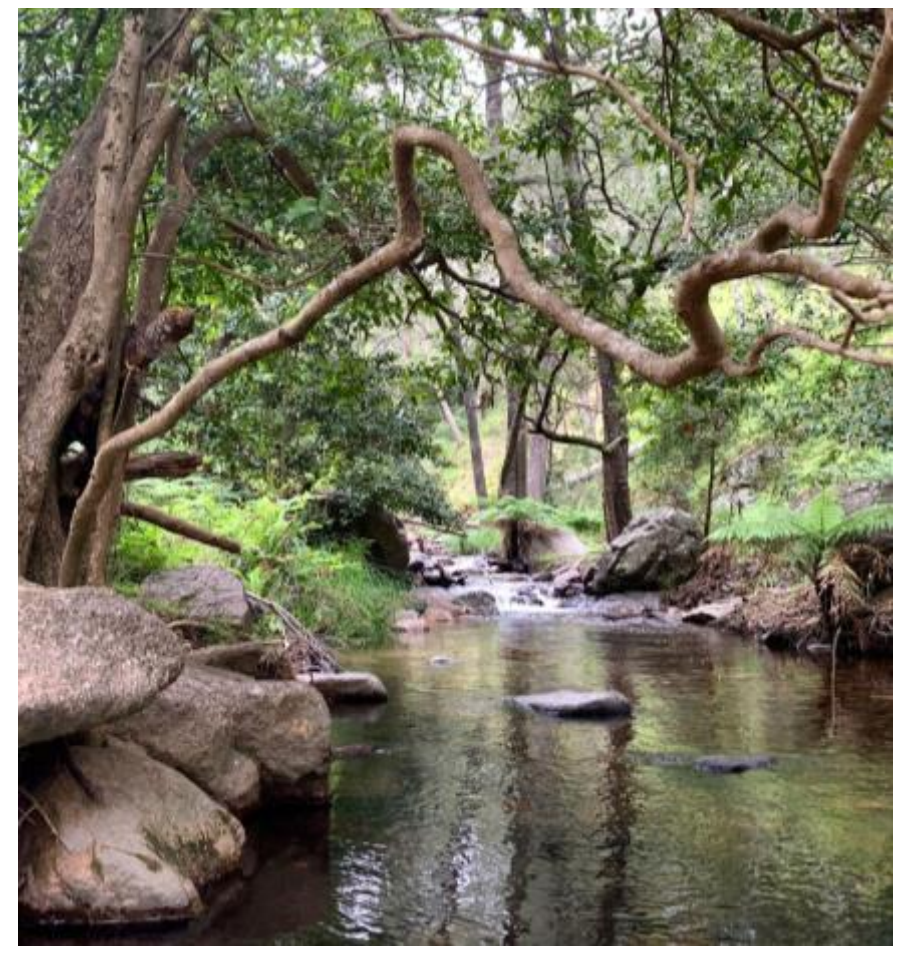
Freshwater Wetland	Low-lying, treeless communities either permanently or temporarily inundated by water. Located on low-nutrient sandy-peat soils. Dominated by sedges, grasses and herbs. Covers 0.4% of the region.	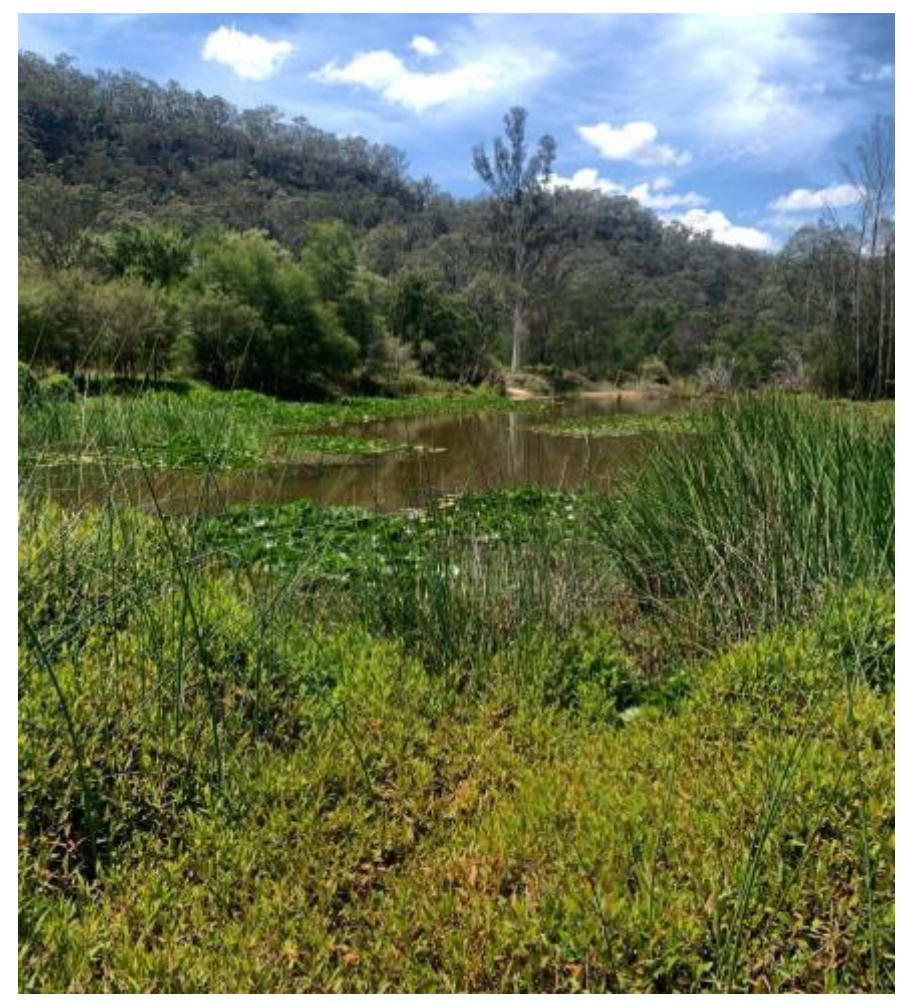

Table S2: Fire severity classifications as described by Gibson et al. (2020). Canopy scorch describes when leaves are killed but not consumed.

Fire Severity	*Description*
Unburnt	Unburnt, green canopy and understorey.
Low	Burnt understorey with unburnt canopy
Moderate	Partial canopy scorch
High	Complete canopy scorch and/or partial canopy consumption
Extreme	Full canopy consumption

Table S3: Model comparison using Akaike's Information Criterion for model selection. Model with yearly moisture lag had the strongest support.

Model	*k*	*Log likelihood*	*AIC*	*ΔAIC*	*AIC Weight*
Yearly moisture lag	12	-235791.20	471608.40	0	1
Seasonal moisture lag	12	-236005.00	472036.00	427.60	0
No moisture lag	12	-236063.40	472152.80	544.33	0

Table S4: Posterior means and 95% credible intervals of all model parameters across study sites in the Greater Blue Mountains World Heritage Area, Australia, 2015-2024. Reference levels for fire severity and vegetation type set as Unburnt and Dry Sclerophyll Forest respectively. Effects considered credible and shown in bold if 95% credible intervals do not cross zero.

Regression coefficients	*Estimate*	*Error*	*Lower 95% CI*	*Upper 95% CI*	*R-hat*	*Bulk ESS*	*Tail ESS*
Main Effects							
Intercept	**22.87**	3.96	**15.15**	**30.63**	1.00	26929	12805
Extreme Fire Severity	**5726.43**	157.43	**5423.12**	**6040.29**	1.00	17644	12454
High Fire Severity	**5062.20**	115.91	**4833.94**	**5285.95**	1.00	21484	12225
Moderate Fire Severity	**2700.89**	99.03	**2507.41**	**2893.61**	1.00	21013	12704
Low Fire Severity	**1192.60**	107.50	**978.92**	**1399.37**	1.00	19641	12431
Forested Wetland	**-24.86**	5.56	**-35.53**	**-14.00**	1.00	36644	11249
Freshwater Wetland	2.34	5.42	-8.30	12.96	1.00	35876	11300
Grassy Woodland	**-16.63**	6.26	**-28.81**	**-4.61**	1.00	32562	11925
Heathland	8.83	5.43	-1.81	19.56	1.00	31745	10267
Rainforest	-9.65	5.47	-20.39	1.06	1.00	33659	11488
Wet Sclerophyll Forest	**-17.03**	5.56	**-28.01**	**-6.19**	1.00	37464	11285
Soil Moisture	**9.44**	3.79	**2.05**	**16.91**	1.00	34041	11995
Two-way interactions							
Extreme Severity x Forested Wetland	12.90	19.89	-25.34	51.93	1.00	24165	11592
Extreme Severity x Freshwater Wetland	-17.60	23.66	-65.03	28.15	1.00	22568	12821

Extreme Severity x Grassy Woodland	25.86	19.45	-12.77	63.99	1.00	26052	12554
Extreme Severity x Heathland	**-37.52**	18.28	**-73.15**	**-1.14**	1.00	24811	12745
Extreme Severity x Rainforest	-1.34	21.06	-42.30	39.82	1.00	26946	12680
Extreme Severity x Wet Sclerophyll Forest	-6.73	19.74	-45.05	32.18	1.00	27097	12052
High Severity x Forested Wetland	-1.03	16.64	-33.49	31.40	1.00	28534	12211
High Severity x Freshwater Wetland	0.27	16.53	-32.11	32.68	1.00	26462	11460
High Severity x Grassy Woodland	14.62	20.08	-24.08	54.20	1.00	27491	12600
High Severity x Heathland	4.86	15.66	-25.73	35.75	1.00	31306	11156
High Severity x Rainforest	0.54	16.82	-32.70	33.52	1.00	25909	12591
High Severity x Wet Sclerophyll Forest	3.74	17.57	-30.37	38.45	1.00	25170	12756
Moderate Severity x Forested Wetland	3.95	18.00	-31.30	38.63	1.00	26730	11247
Moderate Severity x Freshwater Wetland	**-37.29**	16.74	**-70.02**	**-4.37**	1.00	25017	12197
Moderate Severity x Grassy Woodland	24.89	18.33	-10.70	61.01	1.00	25340	12615
Moderate Severity x Heathland	-5.13	15.71	-35.44	26.03	1.00	24661	10964

Moderate Severity x Rainforest	3.42	16.86	-29.27	36.84	1.00	26247	11566
Moderate Severity x Wet Sclerophyll Forest	29.42	17.80	-5.69	63.81	1.00	25022	11736
Low Severity x Forested Wetland	6.64	20.37	-32.93	46.37	1.00	22940	12299
Low Severity x Freshwater Wetland	-35.57	19.47	-73.86	2.68	1.00	22959	12001
Low Severity x Grassy Woodland	27.11	22.17	-16.68	70.33	1.00	20864	12238
Low Severity x Heathland	-16.55	18.69	-52.96	20.26	1.00	26541	11958
Low Severity x Rainforest	-21.69	18.13	-56.93	13.73	1.00	26001	12749
Low Severity x Wet Sclerophyll Forest	-2.64	18.92	-39.78	34.74	1.00	23346	12650
Extreme Severity x Soil Moisture	**82.90**	15.09	**53.35**	**112.68**	1.00	25506	13109
High Severity x Soil Moisture	**80.57**	11.30	**58.51**	**102.90**	1.00	30048	11858
Moderate Severity x Soil Moisture	**31.93**	12.17	**8.09**	**55.67**	1.00	26338	12627
Low Severity x Soil Moisture	1.79	13.28	-24.26	27.86	1.00	28752	11669
Soil Moisture x Forested Wetland	-6.39	5.36	-16.87	4.22	1.00	35308	11574
Soil Moisture x Freshwater Wetland	**-15.87**	5.29	**-26.32**	**-5.47**	1.00	35178	11651
Soil Moisture x Grassy Woodland	-8.17	5.99	-19.98	3.68	1.00	34875	11142

Soil Moisture x Heathland	-4.24	5.18	-14.33	5.96	1.00	33777	13446
Soil Moisture x Rainforest	2.70	5.29	-7.70	13.23	1.00	36024	11971
Soil Moisture x Wet Sclerophyll Forest	-4.27	5.32	-14.56	6.11	1.00	35050	12078
sYear x Extreme Severity	0.13	17.31	-32.45	32.98	1.00	16238	6385
sYear x High Severity	0.59	17.25	-31.36	34.53	1.00	13594	5786
sYear x Moderate Severity	-0.11	19.54	-30.86	31.27	1.00	14248	5194
sYear x Low Severity	-0.03	19.21	-31.77	32.35	1.00	7686	3763
sYear x Unburnt Severity	**650.57**	85.38	**484.14**	**816.75**	1.00	11980	11975
Three-way interactions							
Extreme Severity x Soil Moisture x Forested Wetland	**-41.52**	18.74	**-78.22**	**-4.97**	1.00	29671	11856
Extreme Severity x Soil Moisture x Freshwater Wetland	28.46	22.23	-14.03	72.31	1.00	27015	12165
Extreme Severity x Soil Moisture x Grassy Woodland	**-45.01**	19.17	**-82.69**	**-7.64**	1.00	26559	12404
Extreme Severity x Soil Moisture x Heathland	-8.29	17.97	-44.11	26.77	1.00	27549	12224
Extreme Severity x Soil Moisture x Rainforest	**-66.15**	20.24	**-105.73**	**-26.40**	1.00	29254	12071
Extreme Severity x Soil Moisture x	20.65	19.36	-17.17	58.16	1.00	28389	12177

Wet Sclerophyll Forest							
High Severity x Soil Moisture x Forested Wetland	-1.03	16.64	-33.49	31.40	1.00	28534	12211
High Severity x Soil Moisture x Freshwater Wetland	**-48.14**	15.39	**-78.46**	**-18.10**	1.00	28188	12039
High Severity x Soil Moisture x Grassy Woodland	**-46.96**	18.73	**-83.88**	**-10.62**	1.00	29476	11217
High Severity x Soil Moisture x Heathland	-8.29	17.97	-44.11	26.77	1.00	27549	12224
High Severity x Soil Moisture x Rainforest	**-51.01**	15.62	**-81.67**	**-20.48**	1.00	26236	11975
High Severity x Soil Moisture x Wet Sclerophyll Forest	-28.59	16.66	-61.14	4.07	1.00	27146	11555
Moderate Severity x Soil Moisture x Forested Wetland	-20.91	17.01	-53.95	12.46	1.00	26571	11392
Moderate Severity x Soil Moisture x Freshwater Wetland	**66.75**	16.01	**35.58**	**97.81**	1.00	27838	12099
Moderate Severity x Soil Moisture x Grassy Woodland	**-40.87**	18.09	**-76.28**	**-5.52**	1.00	26971	12796
Moderate Severity x Soil Moisture x Heathland	12.01	15.12	-17.32	42.15	1.00	24682	12130

Moderate Severity x Soil Moisture x Rainforest	-27.97	15.96	-59.22	2.81	1.00	25161	13044
Moderate Severity x Soil Moisture x Wet Sclerophyll Forest	-13.39	17.02	-46.78	20.21	1.00	26486	11957
Low Severity x Soil Moisture x Forested Wetland	-15.35	18.45	-52.10	20.38	1.00	26963	11818
Low Severity x Soil Moisture x Freshwater Wetland	16.82	18.53	-19.78	53.27	1.00	27953	11796
Low Severity x Soil Moisture x Grassy Woodland	**-65.03**	20.15	**-104.03**	**-25.21**	1.00	25926	11116
Low Severity x Soil Moisture x Heathland	30.67	18.45	-5.77	67.04	1.00	28242	12076
Low Severity x Soil Moisture x Rainforest	-32.74	17.28	-65.99	0.70	1.00	28488	11256
Low Severity x Soil Moisture x Wet Sclerophyll Forest	-27.52	18.89	-64.41	9.26	1.00	25879	12223

Table S5: Estimated marginal effects (slope) of soil moisture on the Post-fire Stability Index within each combination of fire severity and vegetation community. Effects considered credible and shown in bold if 95% credible intervals do not cross zero.

Vegetation Community	*Slope*	*Lower CI*	*Upper CI*
Low fire severity			
Wet Sclerophyll Forest	-20.39	-48.23	7.01
Freshwater Wetland	12.31	-14.88	37.90
Dry Sclerophyll Forest	11.34	-14.54	35.44
Heathland	**37.75**	**12.03**	**64.36**
Forested Wetland	-10.55	-35.92	15.34
Grassy Woodland	**-62.06**	**-93.42**	**-32.20**
Rainforest	-18.92	-41.31	5.03
Moderate fire severity			
Wet Sclerophyll Forest	23.62	-1.09	48.60
Freshwater Wetland	**92.29**	**69.42**	**114.17**
Dry Sclerophyll Forest	**41.40**	**19.18**	**64.43**
Heathland	**49.12**	**29.44**	**69.57**
Forested Wetland	14.08	-10.02	39.49
Grassy Woodland	-7.59	-33.65	19.46
Rainforest	15.97	-7.38	38.45
High fire severity			
Wet Sclerophyll Forest	**57.11**	**30.78**	**83.55**
Freshwater Wetland	**126.73**	**102.59**	**152.40**
Dry Sclerophyll Forest	**89.98**	**69.85**	**111.32**
Heathland	**76.68**	**55.46**	**98.04**
Forested Wetland	**35.53**	**13.23**	**58.60**

Grassy Woodland	**35.05**	**4.67**	**64.31**
Rainforest	**41.73**	**17.70**	**65.87**
Extreme fire severity			
Wet Sclerophyll Forest	**108.58**	**79.36**	**138.33**
Freshwater Wetland	**104.74**	**69.17**	**142.89**
Dry Sclerophyll Forest	**92.21**	**64.42**	**121.43**
Heathland	**79.71**	**54.62**	**106.51**
Forested Wetland	**44.36**	**17.33**	**73.18**
Grassy Woodland	**39.14**	**12.15**	**66.07**
Rainforest	28.93	-1.87	59.36

Figure S1: Recovery trajectories over the period 2020 to 2024 of vegetation communities following **low severity fire** (solid arrows) compared to unburnt areas (dashed arrows) in the Greater Blue Mountains World Heritage Area. Values representing vegetation stability are shaded in grey. Bars represent 95% credible intervals.

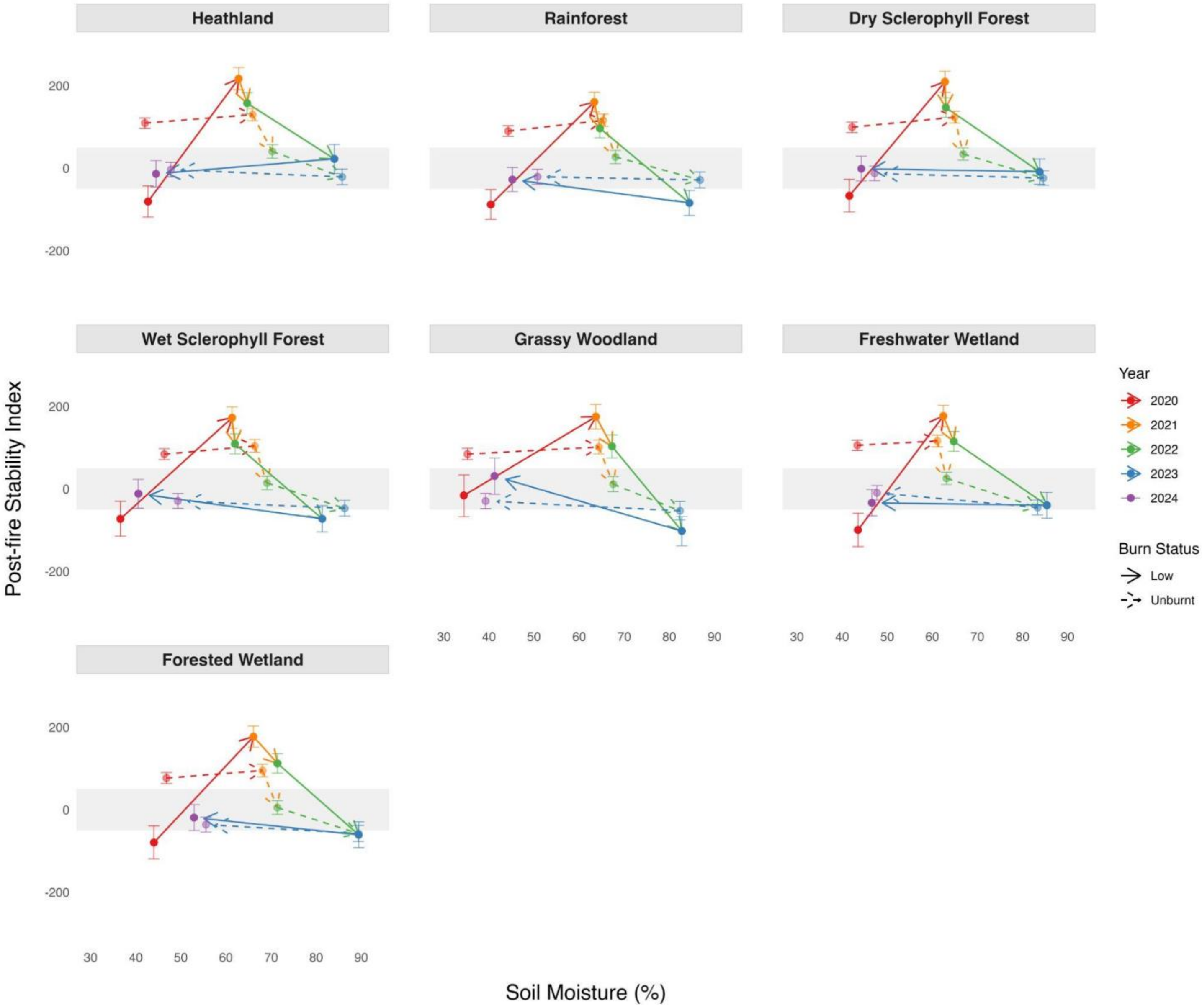

Figure S2: Recovery trajectories over the period 2020 to 2024 of vegetation communities following **moderate severity fire** (solid arrows) compared to unburnt areas (dashed arrows) in the Greater Blue Mountains World Heritage Area. Values representing vegetation stability are shaded in grey. Bars represent 95% credible intervals.

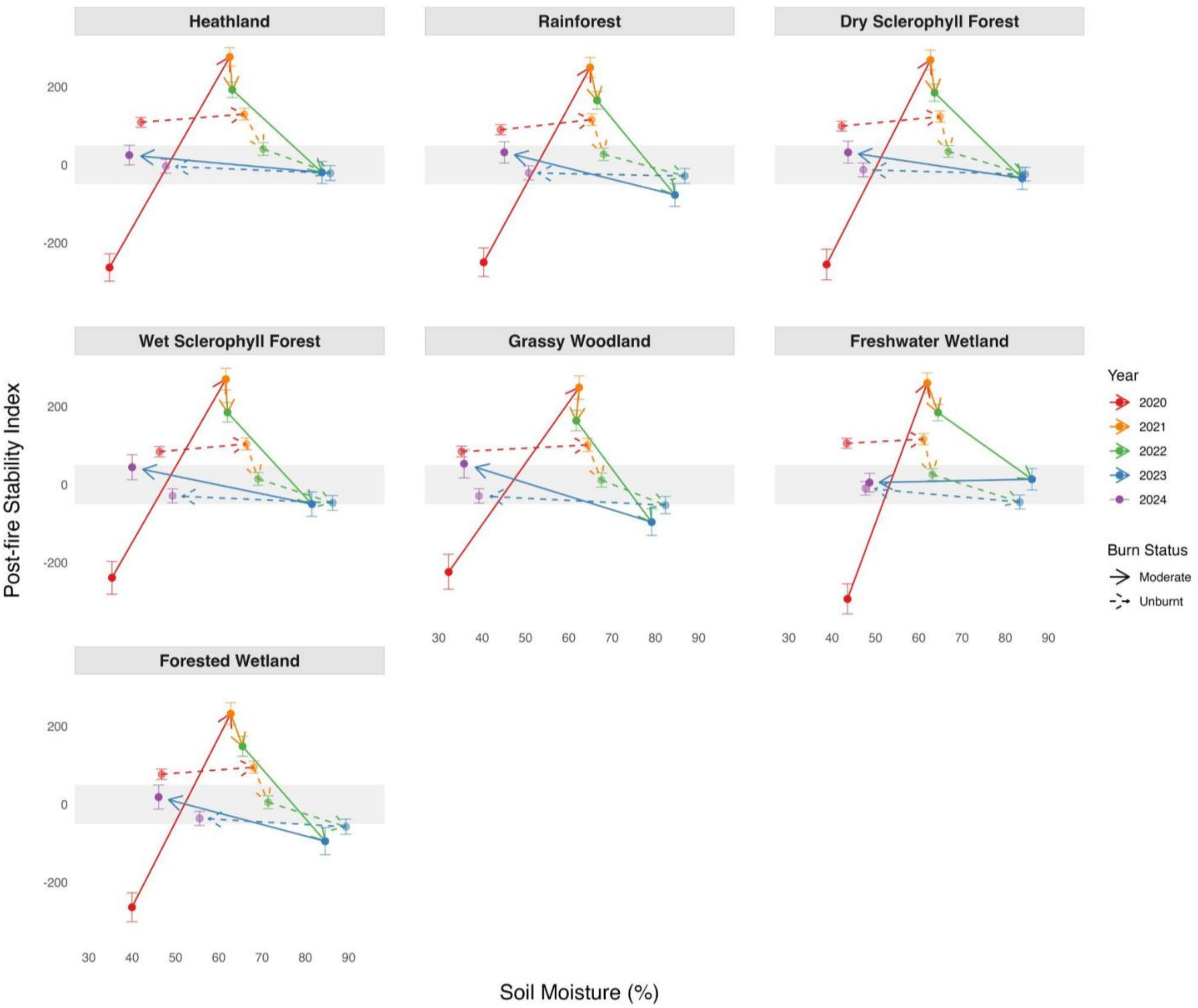

Figure S3: Recovery trajectories over the period 2020 to 2024 of vegetation communities following **high severity fire** (solid arrows) compared to unburnt areas (dashed arrows) in the Greater Blue Mountains World Heritage Area. Values representing vegetation stability are shaded in grey. Bars represent 95% credible intervals.

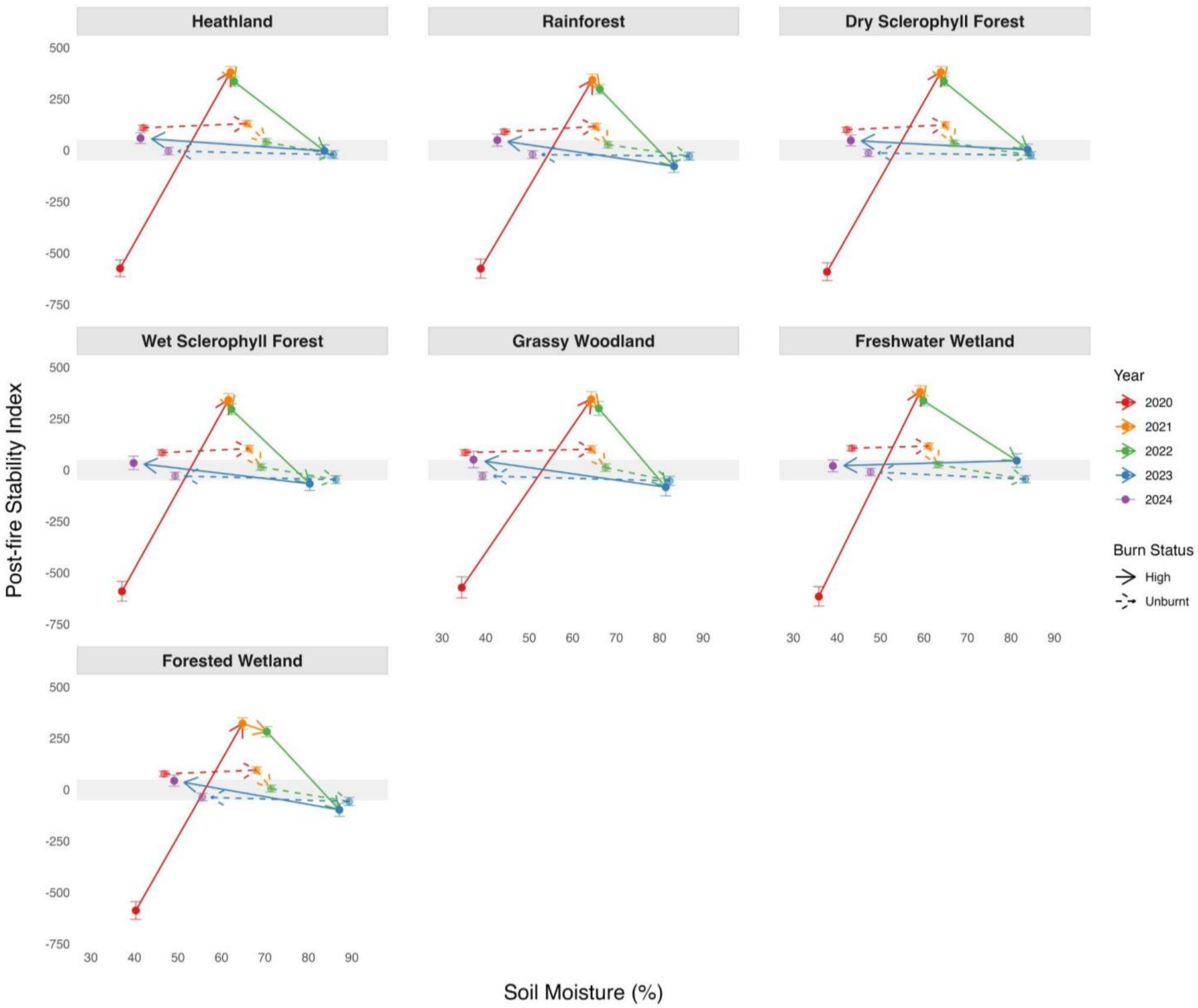

Figure S4: Recovery trajectories over the period 2020 to 2024 of vegetation communities following **extreme severity fire** (solid arrows) compared to unburnt areas (dashed arrows) in the Greater Blue Mountains World Heritage Area. Values representing vegetation stability are shaded in grey. Bars represent 95% credible intervals.

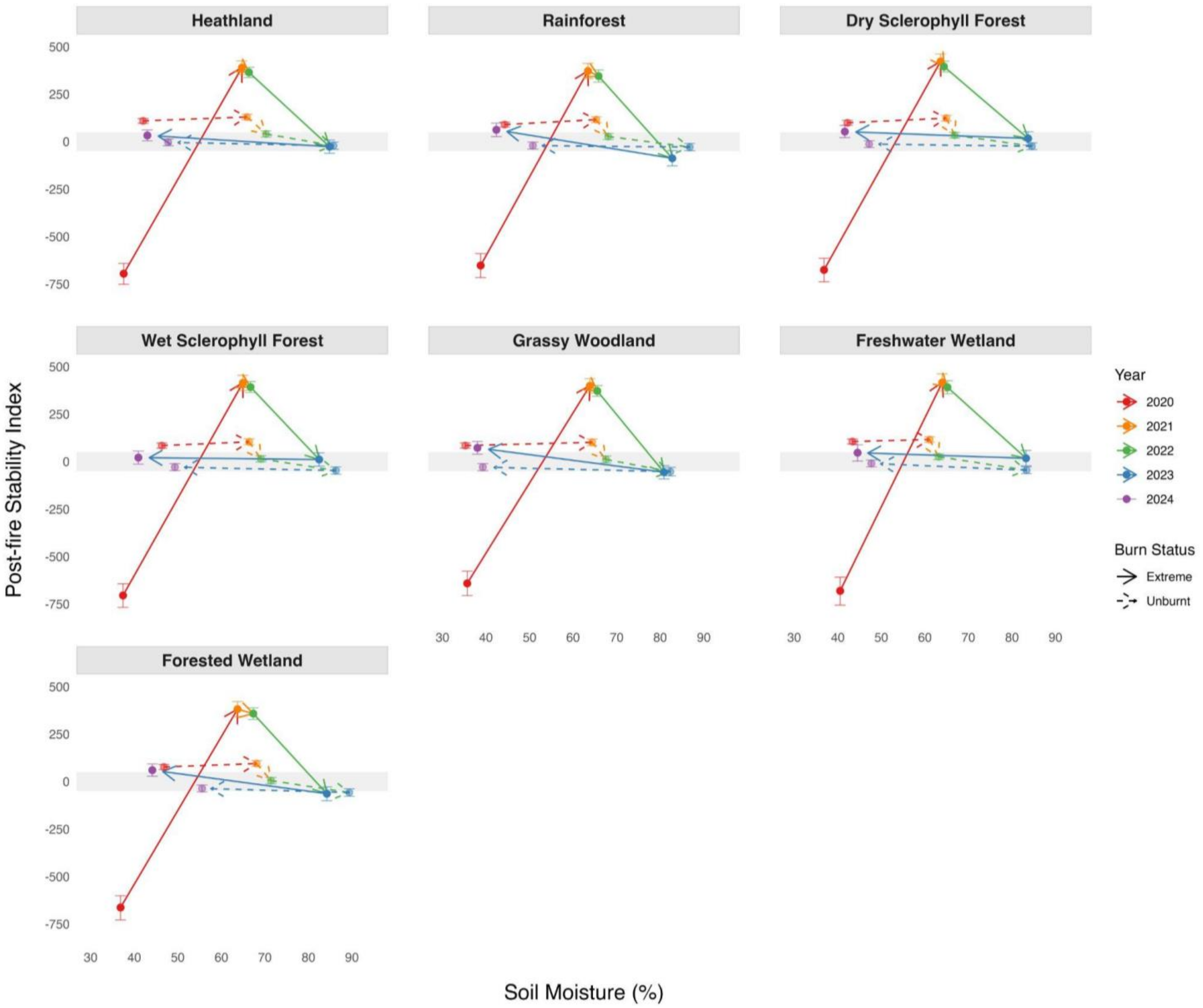

Supplement: Supplementary file 1 [file GCB-32-e71057-s001.pdf]
